# The Origin of The Acheulean: The 1.7 Million-Year-Old Site of FLK West, Olduvai Gorge (Tanzania)

**DOI:** 10.1038/srep17839

**Published:** 2015-12-07

**Authors:** F. Diez-Martín, P. Sánchez Yustos, D. Uribelarrea, E. Baquedano, D. F. Mark, A. Mabulla, C. Fraile, J. Duque, I. Díaz, A. Pérez-González, J. Yravedra, C. P. Egeland, E. Organista, M. Domínguez-Rodrigo

**Affiliations:** 1 Department of Prehistory and Archaeology, University of Valladolid, Pza. del Campus, s/n, 47011 Valladolid, Spain; 2 Department of Geodynamics, Complutense University, c/José Antonio Novás 12, 28040 Madrid, Spain; 3 Museo Arqueológico Regional, Plaza de las Bernardas s/n, 28801 Alcalá de Henares, Madrid, Spain; 4 NERC Argon Isotope Facility, Scottish Universities Environmental Research Centre (SUERC), East Kilbride, Scotland, G75 0QF, UK; 5 IDEA (Instituto de Evolución en África), Museo de los Orígenes, Plaza de San Andrés 2, 28005 Madrid, Spain; 6 Archaeology Unit, University of Dar es Salaam, Dar es Salaam, P.O. Box 35050 Tanzania; 7 Centro Nacional de Investigación sobre la Evolución Humana, CENIEH. Pza. Sierra de Atapuerca s/n, 09002 Burgos, Spain; 8 Department of Prehistory, Complutense University, Prof. Aranguren s/n, 28040 Madrid, Spain; 9 Department of Anthropology, University of North Carolina at Greensboro, Greensboro, USA

## Abstract

The appearance of the Acheulean is one of the hallmarks of human evolution. It represents the emergence of a complex behavior, expressed in the recurrent manufacture of large-sized tools, with standardized forms, implying more advance forethought and planning by hominins than those required by the precedent Oldowan technology. The earliest known evidence of this technology dates back to *c*. 1.7 Ma. and is limited to two sites (Kokiselei [Kenya] and Konso [Ethiopia]), both of which lack functionally-associated fauna. The functionality of these earliest Acheulean assemblages remains unknown. Here we present the discovery of another early Acheulean site also dating to *c*. 1.7 Ma from Olduvai Gorge. This site provides evidence of the earliest steps in developing the Acheulean technology and is the oldest Acheulean site in which stone tools occur spatially and functionally associated with the exploitation of fauna. Simple and elaborate large-cutting tools (LCT) and bifacial handaxes co-exist at FLK West, showing that complex cognition was present from the earliest stages of the Acheulean. Here we provide a detailed technological study and evidence of the use of these tools on the butchery and consumption of fauna, probably by early *Homo erectus sensu lato*.

Large-sized handaxes, cleavers and picks, usually made on large (≥10 cm) flake blanks, but also on cobbles or tabular clasts, are the most widely known tool types of the Acheulean technology^1^,^2^,^3^,^4^,^5^. It has been argued that the Acheulean represents a more complex industry than the previous (and pene-contemporary) Oldowan industry (which consists mostly of small flakes, flaked cobbles and percussive tools) based on the technological ability to produce large flake blanks and to recurrently shape large cutting tools (LCTs)^6^. In regions were both tehcnologies co-exist, it has been argued that Acheulean assemblages exhibit a higher degree of planning and curation than Oldowan assemblages^7^. Recently, it has also been shown that making Acheulean tools require more complex neurophysiological skills than Oldowan tools^8^. For these reasons, the emergence of the Acheulean is of utmost importance in the evolution of early humans, as it is commonly linked to a web of functional, economic, spatial, technological, and cognitive adaptations that boosted human evolution^9^,^10^,^11^,^12^,^13^,^14^,^15^,^16^,^17^,^18^,^19^,^20^,^21^,^22^.

Traditionally, most early Acheulean assemblages show either no association with fossil faunal remains^23^,^24^,^25^,^26^,^27^,^28^,^29^ or if they do, no functional evidence of the association is documented in most of them through taphonomic research^30^,^31^,^32^. This circumstance prompted eco-behavioral models of different activities by hominins and diverse site functionality according to the paleo-geographic and paleo-ecological location of Acheulean and Oldowan sites^27^,^33^. The earliest Acheulean discovered at Konso Gardula (Ethiopia)^23^ and Kokiselei (West Turkana, Kenya)^24^ have further reinforced this view. However, neither site contains fauna functionally associated with their stone tool assemblages. Here we report (geological, chronological, technological and taphonomic analysis) on the discovery of an Acheulean assemblage at FLK West (FLKW), which in contrast with other Acheulean sites, occurs in spatial and taphonomically-supported functional association with abundant fossil fauna. This provides valuable information on the behavioral and technological origins of the Acheulean, by functionally linking some of the artefacts of this technology to carcass butchery. This was previously only feasible with the Oldowan technology for the Early Pleistocene archaeological record. In addition, it was previously argued that the Acheulean technology followed an evolutionary trend of increasing complexity^23^. Here we also show that evolved handaxes, such as those documented <1.4 Ma in Konso were already present in the dawn of the Acheulean technology, implying similar levels of complex cognition.

## FLKW Geology

**Figure 1:**
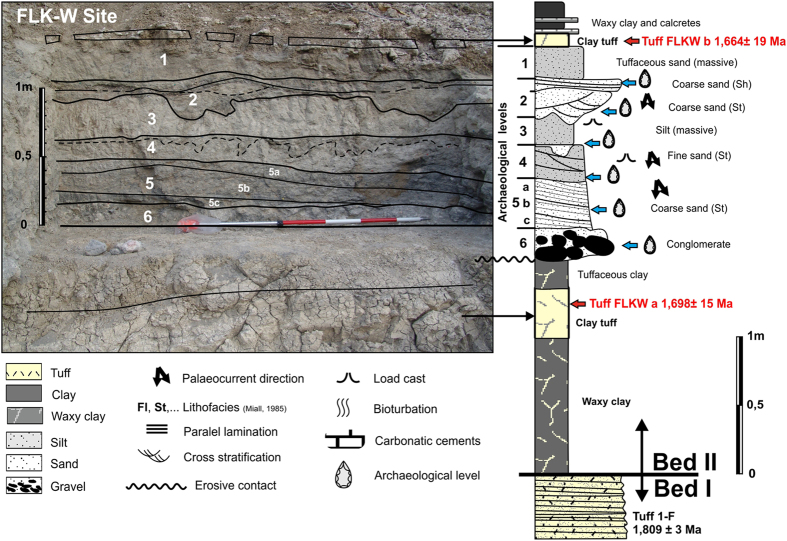
Left, detail of geometry and contacts of geological levels 1 to 6 in FLKW site. Right, stratigraphicsection from Tuff 1-F to Tuff FLKW b. Drawing and photo by D. Uribelarrea.

FLKW, discovered in lowermost Bed II at Olduvai Gorge (Tanzania), is located in a fluvial paleochannel embedded in the clay unit that forms the base of Bed II. The river channel is about 40 m wide with 1.2 m of maximum depth and it is infilled with a sequence of six stratigraphic levels (Fig. 1). Each one corresponds with a flood event, which resulted in a fluvial deposit. The lowermost levels (L5 and L6) are the most dense and important in terms of their archaeological contents.

**Figure 2:**
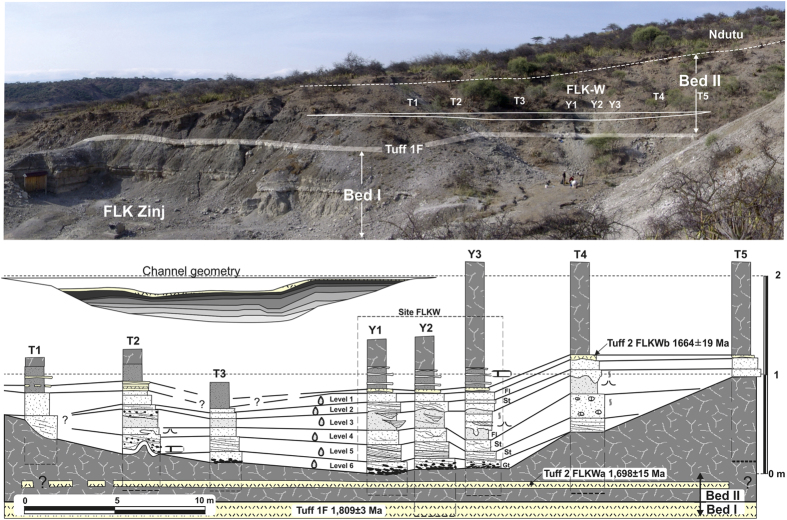
Above, general view of the new site FLKW. T1-5, geotrenches. Y1-3, archaeological trenches. Below,cross-section showing the stratigraphy of the lowermost Bed II and the FLKW channel. Lithological symbols are the same as in Figure 1. Drawing and photo by D. Uribelarrea.

The channel flowed North-South, from the Lemagrut slopes to the central Olduvai paleo-lake. With a lensed and symmetric shape, the deepest part of the channel is about 20 m wide and both margins are shallow (Fig. 2). The complete sedimentary sequence is fining upwards, according with the typical infilling of a fluvial channel. The lowermost level (L6), is a 20 cm thick matrix-supported conglomerate, composed of blocks, cobbles and gravels (150–2 mm of max. diameter), and a matrix of coarse sand. Most of the cobbles are composed of basalt. Although it is a bedload unit, no flow structures, such as cross-bedding or imbricated cobbles have been found. It corresponds with the highest energy level within the fluvial deposit and it is clearly erosive (Fig. 2). Level 5 (L5) is composed by three layers of coarse sand, tilted 10° westwards. The complete sequence is 20–30 cm thick, with a nearly horizontal base and gently undulated top. This bedload is mostly composed of basalt, but there are also quartzite and carbonate pellets. This fluvial bar migrated to the west. Cross bedding and horizontal lamination can be identified in the lower and western parts of sequences 5a, 5b and 5c. Level 4 (L4) is composed of medium-grained sand and well selected fine tuffaceous sands. The thickness of this layer increased to the west, from 15 to 30 cm, adapting to the previous unit 5. Level 3 (L3) is a 30 cm layer of massive clayish silt without flow structures. Level 2 (L2) is an erosive and complex unit, which contains cut and fill shapes up to 50 cm wide and 20 cm deep in the lower part, and interbedded lensed units in the upper section. It corresponds with small braided channels that reworked the previous fluvial deposit. Finally, Level 1 (L1) is formed by massive homogeneous fine-grained sand and silt, overlaid by Tuff FLKWb. All levels are progressively wider, forming an onlap over the river banks. Thus, L1 and L2 can be followed more than 40 m from the central part of the channel (Fig. 2).

This channel represents a high-energy depositional environment, apparently isolated in a wide and flat area. During Bed II, the lake progressively contracted due to increasing aridity. Fresh water availability was reduced and concentrated on fluvial corridors. As a result, hominins also concentrated their activities around river channels, as was documented in the SHK and BK sites, in middle and upper Bed II respectively^34^,^35^,^36^.

## Dating

Single crystals of sanidine were harvested from samples and dated using the 40Ar/39Ar technique at the Scottish Universities Environmental Research Centre. Supplementary information contains a detailed discussion of the methodology but briefly, samples were fused using a CO_2_ laser and the Ar isotope composition of the clean extracted gases was measured using a fully automated MAP 215-50 noble gas mass spectrometer. Ages are reported relative to the optimization model of Renne *et al.* 2011 and the age uncertainty includes decay constant uncertainty.

At the junction of the Main and Side Gorges, uppermost Bed I and the lowermost Bed II are composed of the same waxy clay types, corresponding to a low-energy lake-margin depositional environment. This lake-margin clay is ~6 m thick at FLKW and its surrounding area and marker tuff IF is located in the middle of this clay unit. The last non-reworked marker tuff from Olduvai Gorge, Tuff IF (*c*. 1.8 Ma.)^37^, was deposited in this low-energy setting. Overlaying Tuff IF, a 3-m sequence of waxy clay, similar to that underlying this marker tuff, was deposited. Within these clays, only one meter above Tuff IF, an aeolian yellow tuff, partially transformed into waxy clay is documented (FLKWa). This tuff underlies the FLK W site and it has been dated to 1.698 ± 0.015 Ma (methods and raw data presented in Supplementary Information). The waxy clay that contains it is partially eroded by the FLKW channel and just overlying the channel there is a 30 cm laminated tuff (FLKWb). This tuff has a greater lateral outcropping than FLKWa and has been dated to 1,664 ± 0.019 Ma. These chronological constraints situate FLKW right above Tuff IIA, which was previously dated to *c*. 1.7 Ma^38^,^39^. Hay 1976 highlighted that throughout this region most of Bed II is eroded away by later incision. At locality 45 (FLK) this erosion affected the Lemuta Member and Tuff IIA, creating the first regional discordance identified. The discordance represents a sedimentary change: above the discordance only medium to high-energy sediments were deposited, in contrast with the underlying waxy clays. FLKW is precisely situated at the base of this transition, before the waxy clays give way to detritic and erosive sedimentation of the overlying stratigraphic sequence.

## The stone tool industry

**Figure 3:**
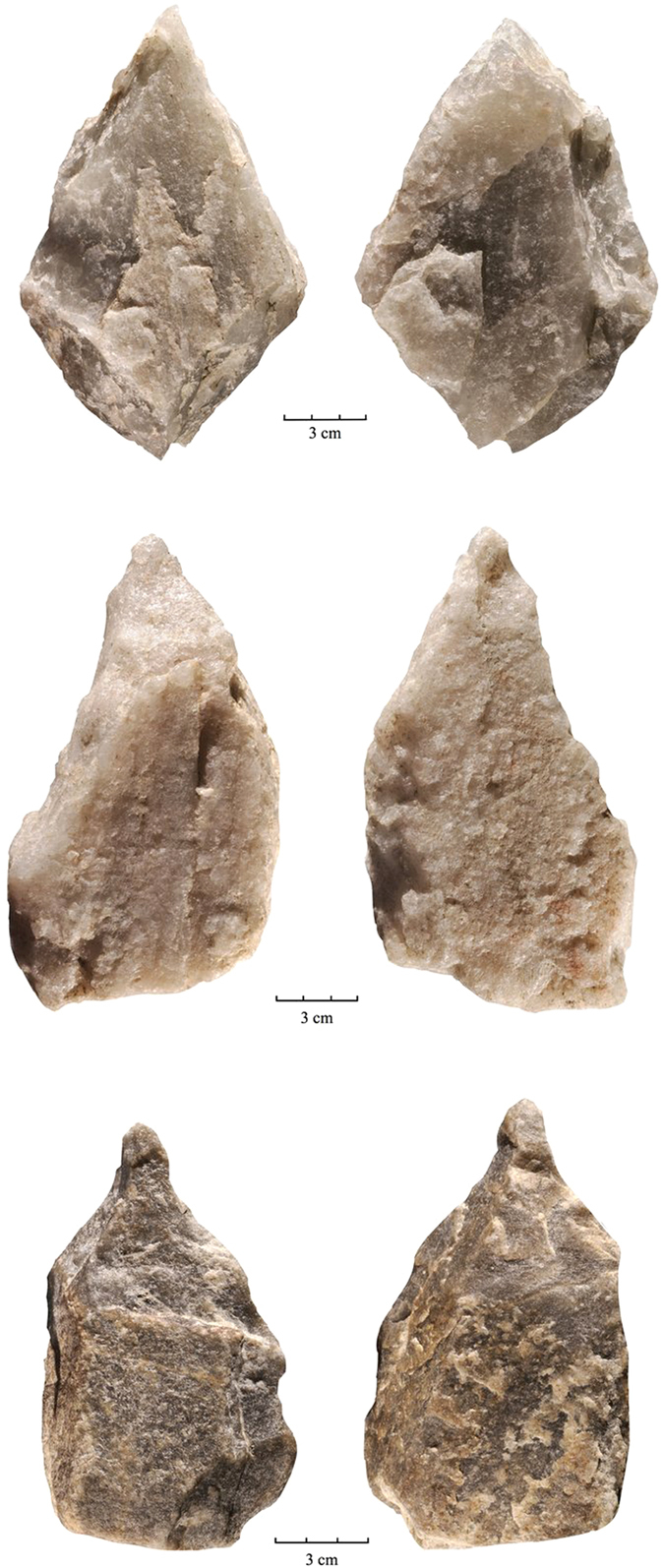
Quartz LCTs recovered from FLKW L5^3^ and L6^1^,^2^. Photo by F. Diez-Martín.

**Figure 4:**
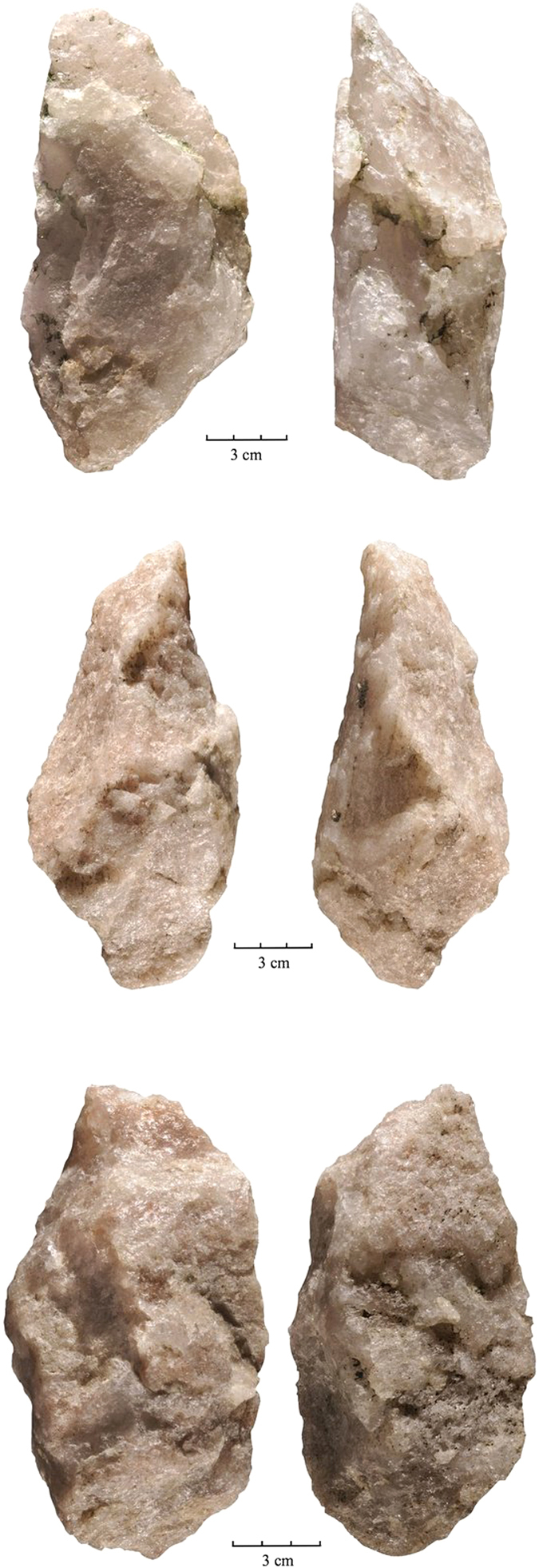
Quartz LCTs recovered from FLKW L6. Photo by F. Diez-Martín.

**Figure 5:**
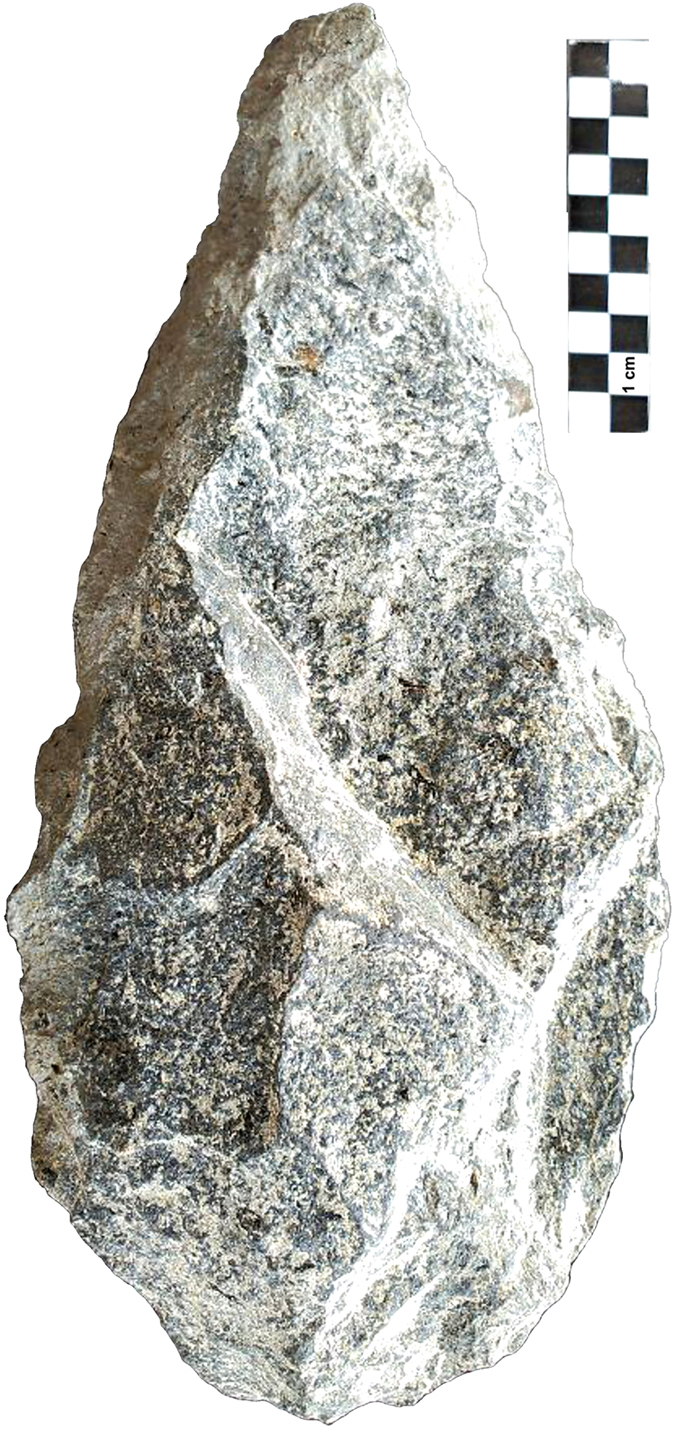
Bifacially-flaked basalt handaxe from FLKW L6. Its dimensions are: 310 mm × 140 mm × 83 mm × 3660 g. Photo by F. Diez-Martín and D. Uribelarrea.

The lithic sample retrieved from FLK West totals 2120 artifacts, distributed throughout the 6 archaeo-stratigraphic units recognized in the excavated sequence (for a more detailed description of the archaeo-stratigraphic interpretation and the technological study of the lithic materials distributed by levels see the Supplementary Information). The bulk of the lithic collection is made from Naibor Soit quartz (73.67%), followed at a distance by volcanic rocks (18.2%) and chert (7.07%). In all stratigraphic levels, a variable representation of unmodified cobbles, percussive elements (complete and fragmented hammerstones and battered cobbles, anvils and percussion flakes), undetermined fragments, and detached objects (<25 mm debris, undetermined shatter and detached residues, freehand and bipolar flakes, and retouched flakes) have been found. Cores for the production of medium-sized flakes mostly follow a variety of unifacial, bifacial and multifacial reduction strategies, among which lineal, orthogonal and centripetal models are represented. Bipolar reduction on anvil is also documented in the sample. From a typological and technological point of view, the most significant Acheulean traits are represented in the two lowermost levels, particularly in L6, where we have documented: 35 large flakes on Naibor Soit quartz (≥100 mm of maximum length, range 100–163 mm), 4 massive cores devoted to the production of large flakes and 28 large cutting tools (LCT), including truly bifacial handaxes. These artefacts are particularly large in maximum length, width and thickness (mean size of 143 × 85 × 53 mm.) and have been knapped mostly on large flake blanks or tabular Naibor Soit clasts. At FLKW, most LCTs have been shaped through bifacial and preferentially non-invasive reduction. Most specimens can hardly be ascribed to the classic Acheulean types (such as hand-axes, cleavers and trihedral picks). Most of them are crude and lack any form of symmetry. However they have been recurrently and efficiently shaped or retouched in order to produce forceful distal pointed areas (*n*11) that on occasions produce formal and massive trihedral picks, combinations of distal points and broad cutting-edges (*n*7), cutting edges (*n*4), and even 2 crude cleavers and 2 knife-like specimens (Figs 3 and 4). These simple LCT forms co-exist in the same assemblage with another extremely sophisticated (highly symmetrical and bifacially flaked) handaxe type (Fig. 5).

To date, the technological traits (i.e., ability to produce large flakes) and typological traits (i.e., ability to produce large and heavy tools with redundant shapes and/or morpho-functional structures) documented in FLKW L5 and L6 are unique for the archaeological record of Olduvai Gorge prior 1.5 Ma^40^,^41^. They are completely in agreement with those traits traditionally ascribed to the Early Acheulean^6^, particularly with the earliest examples recently documented in East Africa for a similar chronological range (*c.* 1,7 M.a). At Kokiselei 4, the Early Acheulean sample is also characterized by the production of large flakes^42^ and by the recurrent shaping of points^24^,^43^. The same emphasis on thick pointed tips and forceful cutting edges has been documented in the earliest Acheulean assemblages at Konso^23^, and Busidima Formation at Gona, dated at *c*.1.6 Ma^44^. However, and in contrast with Kokiselei and Konso, FLK West provides evidence of relatively advanced knapping skills expressed in handaxes that have been bifacially flaked and in which maximum symmetry has been obtained. This shows that complex knapping skills were present from the beginning of the Acheulean stage and that shape variability in LCTs must be accounted for behavioral features not necessarily related to cognition.

## The faunal assemblage

The FLK West faunal assemblage is diverse, but taxonomically dominated by open-habitat taxa (Supplementary Information Table S10). A minimum of 20 individuals have been identified, belonging mostly to Bovidae (namely alcelaphini), Suidae and Equidae. Table S10 (Supplementary Information) shows the distribution of remains and individuals per taxa and archaeological level. Most anatomical elements belong to (by order of abundance) long bones, teeth and axials (ribs). Preservation of remains is good although several surfaces have been affected by exposure to water. The most taphonomically relevant feature is that virtually most of the long bone remains show clear green-broken planes, regardless of carcass size. Bone surface modifications include cut, percussion and tooth marks (Supplementary Information Table S11).

A total of 4 cutmarked bones (3.2%), 13 percussed specimens (10.4%) and 14 toothmarked fragments (11.2%) have been documented, including the bone assemblages from both archaeological levels. All of these specimens bearing marks have been identified on long bone specimens (n = 125). Cut marks occur on the shafts of a humerus, a metatarsal and two tibiae, as is commonly documented on defleshing. Although the number of cutmarked specimens is too ambiguous (given the sample size) to determine if hominins were defleshing carcasses primarily or secondarily, both percussion marks and tooth marks are within experimental scenarios simulating hominin demarrowing of bones followed by carnivore post-depositional ravaging.

The importance of this information is that it provides firm evidence that there is a functional relationship of the stone tool assemblages from levels 5 and 6 with their respective faunal assemblages. This indicates that carcass butchery was one of the activities repeatedly carried out at the site by these early Acheulian hominins (Supplementary Information Figs S24–S29). However, this neither demonstrates nor refutes that LCTs were used in butchery. Future functional study of the tools should show which Acheulian tools were effectively used during butchery.

## Discussion

The oldest Acheulean site at Konso Gardula (KGA6-A1) is bracketed in between two tuffs (1.74–1.66 Ma)^23^. The assemblage is stratigraphically situated much closer to the lower tuff suggesting an age of *c*. 1.7 Ma. The age of Kokiselei 4 is constrained indirectly through regional correlation - the stratigraphy shows that Kokiselei 4 is *c*. 5 m above the Olduvai-Matuyama transition and *c*. 25 m above the *c*. 1.9 Ma KBS Tuff 24. The age of Kokiselei 4 is locally estimated via sedimentation rates within this regional framework. A cubic spline fit to several chronostratigraphic points provided an estimate of *c*. 1.8 Ma^24^; however, the lack of a robust marker horizon in close proximity to Kokiselei 4 and regional correlation to the *c*. 1.5 Ma Lower Koobi Fora Tuff suggests a site age of *c*. 1.7 Ma with an open margin for chronological variation. This age is also consistent with the earliest evidence of Acheulean at Gona (*c*. 1.7 Ma.)^45^. FLK West constitutes one of the earliest chronologically well-bracketed Acheulean sites. Both dates from tuffaceous sediments underlying and overlying the site are extremely close in time and convey confidence for the estimate of c. 1.7 Ma for the Acheulean assemblage from L6.

Following discovery of the two *c*. 1.7 Ma Acheulean sites from Konso and Kokiselei, there existed a c. 0.2 Ma hiatus to the more common Acheulean sites dated to <1.5 Ma. The discovery of FLKW shows that Acheulean technology prior to 1.5 Ma is more common than previously thought. Furthermore, the basic volumetric features that characterize this techno-complex can also be found in assemblages formally and traditionally ascribed to the Developed Oldowan postdating 1.7 Ma^46^. Several technological traits typically linked to the Acheulean concept, such as complex volumetric treatment of medium-sized cores and LCT shaping have also been reported for some Olduvai sites typically classified as Developed Oldowan^46^. The co-existence of a dual typological tool-kit across most of the African Early Pleistocene sequence, although technologically convergent, indicates that different behaviors and site functionality determined the use and accumulation of assemblages previously labelled as Acheulean or Developed Oldowan^6^,^47^. At FLK West (L6) variability in LCT shaping is documented, expressing in some cases complex morphological and technological designs (Fig. 5), which typologically were until now identified in much later assemblages. Similar morphotypes are documented in Konso in assemblages <1.4 Ma^23^. This mix of LCT formats indicates that the morphology of LCTs is not evolutionarily unilineal. It also shows that complex cognition accompanies the earliest stages of the Acheulean.

It must be stressed, in support of this interpretation, that most early Pleistocene Acheulean assemblages at Olduvai Gorge, Koobi Fora, Olorgesailie and Peninj are either devoid of fauna or, when present, fossils are functionally unassociated to stone tools^25^,^26^,^27^,^40^,^48^,^49^,^50^. Even if preservation factors could only partially have accounted for this situation, this certainly is not the case for several of these assemblages (e.g., TK, Peninj)^27^,^51^. FLKW is exceptional in this regard because it contains Acheulean tools associated with abundant fauna. Most early Acheulean sites devoid of fauna makes their functional understanding complicated. Morphological and technological study of these artefacts is insufficient to understand what activities were performed at those sites. Similar cutting or hacking activities could be carried out for a wide diversity of purposes (e.g., butchery, wood working, plant processing, tuber digging). The lack of faunal remains, though, should not hamper interpretations of the functionality of such sites. For instance, phytolith analyses suggested that some handaxes at Peninj were used for wood working^52^. The use of starch, phytolith and biomarker studies could open new ways of understanding activities performed with Acheulean stone tools. Butchery of carcasses associated with Acheulean sites (combining *in situ* and *ex situ* materials) was reported for the Konso area, with convincing evidence after 1.5 Ma^53^. The taphonomic study of FLK West shows that the assemblage is anthropogenic and that hominins engaged in the exploitation of carcasses of various sizes at the site at an earlier date than Konso. The presence of cut and percussion marks as well as the majority of green-broken bone shows that hominins butchered carcasses accumulated at the site. This shows that the Acheulean-Developed Oldowan typological dichotomy cannot be accounted for in behavioral terms at all sites. It also shows that Acheulian sites are behaviourally diverse.

The Acheulean technology has been argued to be the hallmark of *H. erectus.* However, at present this interpretation must be nuanced in the light of hominin types chronologically co-occurring with this and other technologies. First, the presence of *H. erectus* (or *H. erectus*-like) fossils antedate the earliest evidence of this technology by at least 200 Ka (e.g., the 1.9 Ma KNMER 3228 or OH86)^54^, and they occur at a time in which only classical Oldowan is documented. Secondly, there are *H. erectus* remains directly associated with typologically and technologically Oldowan assemblages (e.g., Dmanisi at 1.7 Ma)^55^. Thirdly, the traditional association of classical Oldowan and *H. habilis* from Olduvai Bed I has been challenged by the presence of a *H. erectus*-like hominin (OH 86) at this time^54^. Therefore, at this stage, behavioral diversity may be as important as biology to explain the patchy and environmentally-patterned ocurrence of early Acheulean stone tool industries.

Stable isotopic composition of soil carbonates from fluvial and aeolian sediments at Olduvai Gorge show that major climatic changes towards aridity are documented first around 1.7 Ma (lowermost Bed II) and 1.3 Ma (uppermost Bed II)^56^. This trend towards aridification and landscape openness is not unique of Olduvai Gorge. It is also recorded at the Omo basin and the Busidima Formation^57^. The coincidence in time of these climatic changes and the occurrence of the earliest Acheulean would suggest a climatic trigger for this technological innovation and its impact in human evolution.

## Additional Information

**How to cite this article**: Diez-Martín, F. *et al.* The Origin of the Acheulean: The 1.7 Million-Year-Old Site of FLK West, Olduvai Gorge (Tanzania). *Sci. Rep.* **5**, 17839; doi: 10.1038/srep17839 (2015).

## Supplementary Material

Supplementary Information

Supplementary Dataset 1
