# Supplementary material for: The Origin of The Acheulean: The 1.7 Million-Year-Old Site of FLK West, Olduvai Gorge (Tanzania)
Source: Sci Rep. 2015 Dec 7;5:17839. doi: 10.1038/srep17839 (PMC4671088; doi:10.1038/srep17839)
Supplement: Supplementary Information [file srep17839-s1.pdf]

THE ORIGIN OF THE ACHEULEAN: THE 1.7 MILLION-YEAR-OLD SITE OF FLK WEST, OLDUVAI GORGE (TANZANIA).

SUPPLEMENTARY MATERIAL

Diez-Martín, F.[1], Sánchez Yustos, P.[1], Uribelarrea, D.[2], Baquedano, E.[3,5], Mark, D.F.[4], Mabulla, A.[6], Fraile, C.[1], Duque, J.[1], Díaz, I.[1], Pérez-González, A.[7], J. Yravedra[8], Egeland, C.P.[9], Organista, E.[8], Domínguez-Rodrigo, M.[5,8*]

[1]*Department of Prehistory and Archaeology, University of Valladolid, Pza. del Campus, s/n, 47011 Valladolid, Spain.*

[2]*Department of Geodynamics, Complutense University, c/ José Antonio Novás 12, 28040 Madrid, Spain.*

[3]*Museo Arqueológico Regional, Plaza de las Bernardas s/n, 28801 Alcalá de Henares, Madrid, Spain.*

[4]*NERC Argon Isotope Facility, Scottish Universities Environmental Research Centre (SUERC), East Kilbride, Scotland, G75 0QF, UK*

[5]*IDEA (Instituto de Evolución en África), Museo de los Orígenes, Plaza de San Andrés 2, 28005 Madrid, Spain.*

[6]*Archaeology Unit, University of Dar es Salaam, Dar es Salaam, P.O. Box 35050 Tanzania.*

[7]*Centro Nacional de Investigación sobre la Evolución Humana, CENIEH. Pza. Sierra de Atapuerca s/n, 09002 Burgos, Spain.*

[8]*Department of Prehistory, Complutense University, Prof. Aranguren s/n, 28040 Madrid, Spain.*

[9]*Department of Anthropology, University of North Carolina at Greensboro, Greensboro, USA*

*m.dominguez.rodrigo@gmail.com

The archaeological excavation of FLK West

FLK West is one of the fertile archaeological outcrops located in the Frida Leakey Korongo site complex, situated a few meters to the West of the main FLK site (Hay 1976, Leakey 1971) (**Figure S1**). Beds I, II and Ndutu sediments outcrop to the West of the FLK gully (**Figures 1 and 2, main paper**). In 2012, in the course of a survey program focused on the Lower Bed II outcrops of the junction area, The Olduvai Paleoanthropological and Paleoecological Project (TOPPP) discovered a number lithics and fossil bones eroding from a gravel horizon located ~1.5 m above Tuff 1F. Some artefacts and bones still retained part of the greyish sands characteristic of what we now know as FLKW Level 6. Among the lithic implements, we could recognize a few specimens that seemed to be technologically and typologically at odds with what it was expected for the archaeological record of Lower Bed II (Acheulean-like large flakes and cutting tools) (Leakey 1971). In order to horizontally reach what seemed to be the source of the eroding materials and to establish the nature of the stratigraphic correlation with Tuff 1F, we opened a 4.8 m^2 test pit with the main goals of describing the stratigraphic sequence in the area and confirming the lowermost Bed II context for the archaeological materials (**Figures S2 and S3**). For the excavation of the pit we proceeded following artificial 10 cm spits and recording the materials related to each spit. At the base of the channel, the area we had visually identified as the source of the eroding materials, data were recorded with a total station.

In 2013 we undertook a detailed archaeological excavation in an area of 12 m^2 adjacent to the 2012 test pit (**Figures S2 and S3**). In order to contextualize the archaeological site we also opened six geo-trenches around the archaeological site (**Figures S1 and S4**). Features with spatial meaning of geological, archaeo-stratigraphic, and archaeological nature were recorded with the total station (TOPCON-GPT3015N) (including ~3500 topographic points). For the micro-spatial analysis of the horizontal associations of stone tools and bones in the different archaeo-units we also undertook the photogrammetric reconstruction of synchronic horizontal planes along the sequence (**Figures S5 and S6**). Due to the extremely high density of archaeological materials in the lowermost levels (5 and 6), and in order to ensure a detailed record of data, we eventually decided to reduce the excavation area to ~5 m^2 (**Figure S3**). The lithic

collection studied for this work belongs to the artefacts recorded with the total station in 2012 in Level 6 and those retrieved from the whole 2013 excavation.

Archaeo-stratigraphic analysis of FLK West

With the goal of obtaining a high-resolution archaeological stratigraphy in FLK West, we have carried out an exhaustive archaeo-stratigraphic analysis. A similar procedure is frequently being used for the study of a variety of archaeological sites (Canals et al. 2012, Obregón 2012, Sahnouni et al. 2002, Sañudo and Fernández 2007, Vaquero et al. 2015), following the geological analysis and prior to any study of the archaeological material. The archaeo-stratigraphic analysis allows individualizing groups of items that show high synchronicity, considerably reducing the risk of mixing remains from different occupational events. Although this methodology was conceived at first to be applied to deposits with a homogeneous composition (Canals 1993), it has already shown satisfactory analytical results in those sites with differentiated litho-stratigraphic levels (Diez-Martín et al. 2014).

As the density of archaeological materials significantly differs along the excavated section, it has been necessary to undertake two different series of projections with different scales of reference. First we chose to work with 25 cm depth spits, obtaining a total number of 46 profiles covering the whole study area (10 longitudinal and 36 transversal). This specific interval appeared to be the most suitable to correctly demarcate the limits of the uppermost excavated archaeo-levels (L1-4). However, it seemed to be inappropriate for the analysis of the lowermost units due to the significantly higher archaeological density and, thus, the difficulty to delimit with precision archaeological disruptions between levels. Therefore, it was necessary to make a second series of projections focused on the central part of the study area. By reducing the interval between profiles to 10 cm, it was possible to produce 50 projections (20 of then set longitudinally and 30 transversally).
The whole set of projections produced was analyzed with ArcGis 10 software. By the control of longitudinal and transversal spit intersections (Canals 1993) (**Figure S7**) we have been able to recognize five sterile and continuous horizons within the whole excavated area (**Figure S8**). Depth and homogeneity of these sterile levels is not homogeneous, although all cases coincide with stratigraphic ruptures. The identification

of these five disruptions has made it possible to recognize a total of six archaeo-stratigraphic units, correlatively numbered from top to bottom, and coincident with the stratigraphic units of the fluvial environment (**Figure S9**). All archaeo-units, excepting unit 5, represent the minimum analysis units that can be distinguished in this archaeological deposit. The archaeological materials included in each of these units, therefore, provide a high level of synchronicity. Unit 5, on the other hand, constitutes a micro-palimpsest and could be subjected of further divisions in more detailed archae-stratigraphic analyses.

The FLK West lithic collection

The lithic collection unearthed at FLK West totals 2120 artifacts, distributed unevenly throughout six archaeo-stratigraphic units. The most significant accumulations of stone tools occur in levels 6 (39.38% of the total sample), 5 (37.12%), and 4 (13.49%). **Table S1** shows the distribution of lithics sorted by archaeological level and raw material type. The majority of the collection is made on Naibor Soit materials (quartz and quartzite sum 74.51% of the studied sample), followed at great distance by various types of volcanic rocks (18.19%, including generic basalts, vesicular basalt, and phonolite). In line with the availability of chert outcrops in the Basin between Tuffs 1F and 2B (Kyara 1999), chert represents a not negligible 7.07%, and is particularly abundant in level 6 (41% of chert specimens have been retrieved from this lowermost level). Gneiss constitutes a residual rock type in the studied collection.

We have sorted a sample of 846 specimens (cobbles and percussion tools, fragments, cores, and choppers), accounting for the 40% of the collection, within four categories of abrasion and roundness. While R0 is representative of fresh specimens without signs of polishing on ridges and surfaces, R3 includes those items with severe signs of polishing. **Table S2** shows that, despite being preserved in a fluvial context, almost 90% of the analysed sample shows no traces of polishing and abrasion related to fluvial traction. Specimens classified in categories R2 and R3 are more abundant in the lowermost levels, in agreement with a more energetic fluvial context: 6% of the artefacts in both L5 and L6 can be included in R2 or R3. However, these numbers show, at least, a very limited effect of fluvial traction in the preservation of the whole lithic collection.

Table S3 displays the distribution of artefacts sorted by archaeological level and lithic categories. Natural cobbles, mostly oval in shape, and with no apparent sign of anthropogenic use or transformation represent 7% of the collection (n=150). Most of the specimens included in this category are volcanic materials (95.2%, distributed as follows: basalt, 77.3%; vesicular basalt, 10.6%; phonolite, 7.3%), while only 4.6% are quartz/quartzite nodules. In this group we have included 8 fractured cobbles possibly related to percussion accidents, although their inclusion in the percussive elements group is unwarranted, as no percussion stigma have been observed on their surfaces. Other 7 specimens show scarce and unclear negative scars that could probably be the result of exploitation processes.

We have identified a total of 120 tools within the percussion category: 97 hammerstones, 2 anvils, and 21 spheroids/subspheroids. Hammerstones replicate the same oval shape and preference for volcanic rocks observed in natural cobbles (77 have been made in basalt, 10 in phonolite, and 5 in vesicular basalt, while only 4 are quartz and 1 gneiss). Boxplot in **Figure S10** compares metrical values (maximum length, breadth, and thickness) in natural cobbles and hammerstones, showing a significant size and shape overlapping between both groups. Most hammerstones show pitting preferentially concentrated on one (55%) or two (27%) areas. Only 10 specimens show signs of scaring and mass loss due to percussion. We have documented 2 anvils, one in L5 (a basalt cobble with a flat surface showing abundant pitting), and another in L5 (a quartz tabular block with intense crushing on flat surfaces and ridges). Finally, we have included 21 quartz spheroids and sub-spheroids within the percussive tools, following our own experimental observations on the connection between this classical type and the operational sequence of modified battered blocks on quartz (Sánchez Yustos et al. 2015).

Irregular fragments and shatter (n=358) account for 16.88% of the lithic sample. Most of these objects are quartz specimens (n=290, 81%), followed by volcanic materials (n=38, 10.61%) and chert (n=29, 8.1%). In line with the abundance of quartz waste in the collection (Diez-Martín et al. 2011), most fragments have been interpreted as undetermined positives (detached fragments or flakes) (n=230), although we have also identified 104 core fragments, 7 hammerstone fragments, 4 fragments of spheroids/subspehroids, a 1 LCT fragment.

We have retrieved a collection of 213 cores from FLKW , 64 of which (30%) come from L5 and 115 (53.9%) from L6. Cores have been produced on quartz (n=147, 69%), basalt (n=42, 19.7%), phonolite (n=13, 6.1%), chert (n=9, 4.2%), and gneiss (n=2, 0.9%). Regarding the type of blank selected for undertaking the reduction processes of cores, there is a clear difference between quartz and volcanic rocks. In quartz, tabular slabs or blocks predominate (61%), followed by cobbles (34%), flakes (3.8%) and fragments (0.95%). In volcanic rocks, including basalt and phonolite, cobble blanks represent 92.4%, followed by flakes (3.7%), blocks (1.8%), and fragments (1.8%). Besides the different types of blanks selected by raw material type, a pairwise MANOVA analysis (using the Pillai test) shows that quartz and volcanic cores are similarly sized (p=0.12), and differences are documented when comparing chert to volcanic (p=<0.001) and quartz (p=<0.001) materials.

However, raw material type accounts for a significant variation regarding flake productivity in cores. Mean number of negative scars per quartz core is 7.31, while it is 4.72 in volcanic rocks. **Table S4** shows the distribution of core types sorted by archaeological level. Bipolar knapping on anvil has been identified in 33 cases (15.49% of the core sample), most of them retrieved from L6 (60.6%). As it usually happens with bipolar technique (Diez-Martín et al. 2009, 2010, 2011, Sánchez-Yustos et al. 2015), all specimens included in this group are quartz tabular slabs. About 39% of these cores show intense signs of crushing on platforms and ridges. Bipolar reduction has produced abrupt (90º) and lineal fracture planes in 16 cases. In another 14 pieces, lineal fracture planes tend to arrange in a circular or semi-circular manner, showing core rotation in the same plane. The sum, 3 specimens, shows blank rotation in at least two different planes. Leaving aside test cores (specimens in which one or two non-related negative scars have been observed) and exhausted cores, feehand knapping for the production of small and medium-sized flakes (n=127), tend to show, in this order, bifacial (41.73%), unifacial (29.1%), and trifacial/multifacial (29.1%) reduction strategies. In both unifacial (81%) and bifacial (57%) cores, removals tend to be produced in a sub-parallel way, so negative scars are preferentially lineal. Orthogonal and centripetal patterns are less common, although in bifacial cores both patterns are well represented. Trifacial (n=4) and multifacial (n=33) cores show arrangement of negative scars in three or more planes. Many of these cores would fit within the classical type of polyhedrons. When we look at the reduction strategies sorted by raw

material type, basalt, phonolite and chert tend to be better represented by unifacial or bifacial lineal cores, while most unifacial/bifacial orthogonal and multifacial, and all centripetal patterns are related to the exploitation of quartz. Six cores for the production of large flake blanks have been retrieved from the two lowermost levels (3 quartz and 3 basalt specimens). In all these specimens, at least one negative scar with a maximum length ≥10 cm has been counted. Mean size of these large flakes is 114x 89 mm. In most cases, these cores reproduce bifacial lineal (n=3) and orthogonal (n=1) patterns, although unifacial lineal (n=1) and multifacial (n=1) patterns have also been recorded. No specific core preparation technique has been followed to detach the large flake. In most cases, the large flake is related bifacially or multifacially with other small and medium-size flake series. Only on one occasion, the negative scar of a previously removed large flake has been used as percussion platform for the production of a second large flake on the other side of the block (following a unipolar bifacial reduction model).

We have identified 1248 detached objects in the studied collection. Most pieces have been produced from quartz (n=1074, 86%), followed by chert (n=112, 8.97%), basalt (n=40, 3.2%), quartzite (n=12, 0.96%), and phonolite (n=10, 0.8%). **Table S5** shows the distribution of the different groups of objects included in this category, sorted by level. Half of the collection (n=631) consists of debris ≤ 25 mm (mean maximum length of 16.5 mm). The bulk of debris have been produced mainly in quartz (n=562, 89%) and secondly in chert (n=61, 9.6%), while only 5 specimens (0.79%) correspond to volcanic rocks. It is remarkable that most debris have been recovered from the two lowermost levels, precisely the stratigraphic units where the fluvial channel shows its higher energy. Certainly, debris represents 39% of the total sample retrieved from L5 and 19% from L6, showing the significant preservation of the small lithic fraction at the base of the channel. We have also counted 112 undetermined positives ≥25 mm, preferentially made from quartz (n=194, 93%) and, to a certain extent, related to bipolar reduction of quartz slabs. Clear bipolar flakes, with evident signs of bipolar load application (Diez-Martín et al. 2011) have also been counted in this group (n=23). We have also recognized 13 percussion flakes (11 basalt, 1 phonolite, and 1 quartz), mostly recovered from levels 5 (n=3) and 6 (n=7). Regarding plain flakes, knapping accidents have been common, well in agreement with the preponderance of quartz in the sample (Diez-Martín et al. 2011). Fractures affect 40.32% of plain flakes (counting here 90 flake

fragments and 83 fractured flakes). The more abundant types of fractures recorded are representative of quartz knapping: longitudinal (63%), distal (11.48%), proximal (8.8%), and transversal (6%).

Whole flakes sum 256 specimens. Their raw material is mostly quartz (n=203), followed by chert (n=24), basalt (n=21), quartzite (n=5), and phonolite (n=3). Whole flakes tend to be medium-sized (mean maximum length 38.39 mm), although there is a small group of 9 specimens on the verge of being considered large flakes (max. length range 85-94) (**Figure S11**, **Table S6**). Among the group of whole flakes, we have recognized 5 core flakes (3 in quartz and 2 in basalt). Regarding the technical traits observed in complete flakes, most specimens retain little or no cortex on dorsal areas and butts (Toth's types VI and V), exhibit plain striking platforms, and lineal or orthogonal dorsal arrangements (**Figures S12, S13, and S14**). These general patterns vary slightly when the flake collection is sorted by raw material type (**Table S7**). Quartz and basalt flakes are better represented by plain butts and lineal dorsal patterns, while chert flakes reproduce bi- and multi-faceted striking platforms (21%) and orthogonal dorsal patterns (58%) in higher numbers. Centripetal dorsal patterns are more representative of quartzite flakes (40%) and phonolite flakes (33%) than of chert (8%) or quartz (3%) flakes. Retouched flakes (n=27) represent 2% of the detached objects category. Retouch has been performed on chert (49%), quartz (44%), and basalt flakes (7%). In FLKW, retouched specimens tend to be slightly smaller and thinner than plain flakes (**Table 6**), although they tend to reproduce the same technical patterns observed in plain flakes, such as preference for plain butts, lineal or orthogonal dorsal patterns, and scarce retention of cortex (Toth's types V and VI). The identified types, preferentially through marginal, direct or inverse, and semi-abrupt retouch, are pointed tips (4 awls and 2 becs), denticulates (4), scrapers (4), notches (3), and a varied group of non-organized retouched segments that are difficult to include within a traditional typological list (n=10).

Finally, the detached products category includes a collection of 36 large flakes (≥10 cm), retrieved mainly from L6 (n=34), although we have also documented two large flakes recovered from L5 and L4, respectively. These specimens are significantly larger and wider than the average small and medium-sized plain and retouched flakes commonly recovered from FLK West (**Table S6**). All large flakes have been produced

in quartz. Only six specimens show clear or diffuse signs of bulb of percussion, although all large flakes show a very thick striking platform (mean 63 x 32 mm), preferentially plain (n=17, 47%), followed by cortical (n=9, 14%), removed (n=6, 9%), lineal (n=2, 3%) and broken (n=2, 3%). As it happens in other early Acheulean collections (Diez-Martín et al. 2014a, b), when it has been possible to identify, large flakes tend to show orthogonal arrangements on their dorsal patterns (n=11), although we have also documented lineal (n=3) and centripetal patterns (n=1).

Regarding shaped tools, we have recognized a meager collection of unifacial (n=3) and bifacial (n=2) choppers, in which we have identified a quite continuous distal (n=4) or side (n=1) cutting edge. Four choppers have been shaped on medium quality basalt cobbles and one is a quartz block. To date, a collection of 26 large cutting tools (LCT) has been unearthed from FLK West. The bulk of these LCTs were retrieved from L6 (n=24), while 2 specimens have been retrieved from L5. Regarding raw material type, most LCTs have been made on quartz (n=23), followed by basalt (n=2) and phonolite (n=1). Among those pieces shaped on quartz, 10 have been produced on flake blanks, six of which are significantly large flakes (maximum length range 122-180 mm), and the remaining two medium-sized flakes (max. length >80 mm). The other 13 quartz LCTs have been shaped on large tabular slabs (with a max. length of 216 mm). Blanks of LCTs on volcanic rocks are 2 cobbles (max. length 72-74 mm) and 1 basalt large flake (max. length 108 mm). Regarding the way these LCTs have been shaped, it is very difficult to systematically classify the diversity of shaping strategies observed in formal types (**Figures S15-S23**). We have decided to sort LCTs in morpho-functional groups, following the principle of techno-functional units described by Boëda (2001). A common trait of the majority of these tools is that they have been partially and superficially transformed via marginal and sinuous, abrupt, semi-abrupt or simple retouch, displaying shaping gestures seldom bifacial and invasive. An incipient treatment of volumes and forms related to the basic principles of bifacial symmetry is mostly absent from the LCT collection, although in the few cases where invasive series of bifacial reduction have been described, an incipient bilateral symmetry can be recognized (**Figure S19**). Rather, quite simple shaping processes efficiently operate to produce two basic morphological features with a purported functional meaning that, in occasions, may appear combined in the same implement: pointed areas or broad cutting edges (**Table S8**). Pointed areas, constitute the more abundant feature (n=11, 42.3%). In

this group we include implements in which unifacial, bifacial, or even trifacial work has been focused on the production of distal tips. We have documented examples in which knapping on three surfaces has produced powerful, and clearly highlighted from the basal area, distal points (formal trihedral picks) (**Figure S18**). Other group shows the combination of more or less ample side cutting edges with distal pointed areas (n=7, 27%) . In 3 cases, two unifacially retouched areas converge in a distal tip, while in other 4 cases, one side or oblique retouched area (exhibiting bifacial retouch only in one case) is associated with a distal tip. A third group is represented by specimens in which bifacial and marginal retouch have produced distal and sinuous cutting edges (n=4, 15.4%) (Figure S21.1). Finally, we have included 4 specimens (15.4%) in more traditional types. Two of them have been classified as crude and primitive cleavers (in which a transversal cutting edge is associated to a side retouch) (Figure S21.2). The other two specimens,at least formally, resemble the knife type, in which an abrupt retouched edge is opposite to a cutting edge (Figures S16 and S19).

Taphonomic information

The zooarchaeological and taphonomic information presented here will be in the form of numeric summary. A more detailed analysis will be published in a separate work. The FLK West faunal assemblage is diverse and represents a mostly open-habitat ecosystem (**Table S9**). The taphonomic information (**Table S10**) shows that the assemblage was green-broken and bones preserve cut and percussion marks, which together with the low frequency of tooth marks suggest hominin exploitation of a diverse number of carcasses (**Figures S24-29**), including megafauna. This makes FLK West the oldest Acheulean site with evidence of butchery.

FIGURES

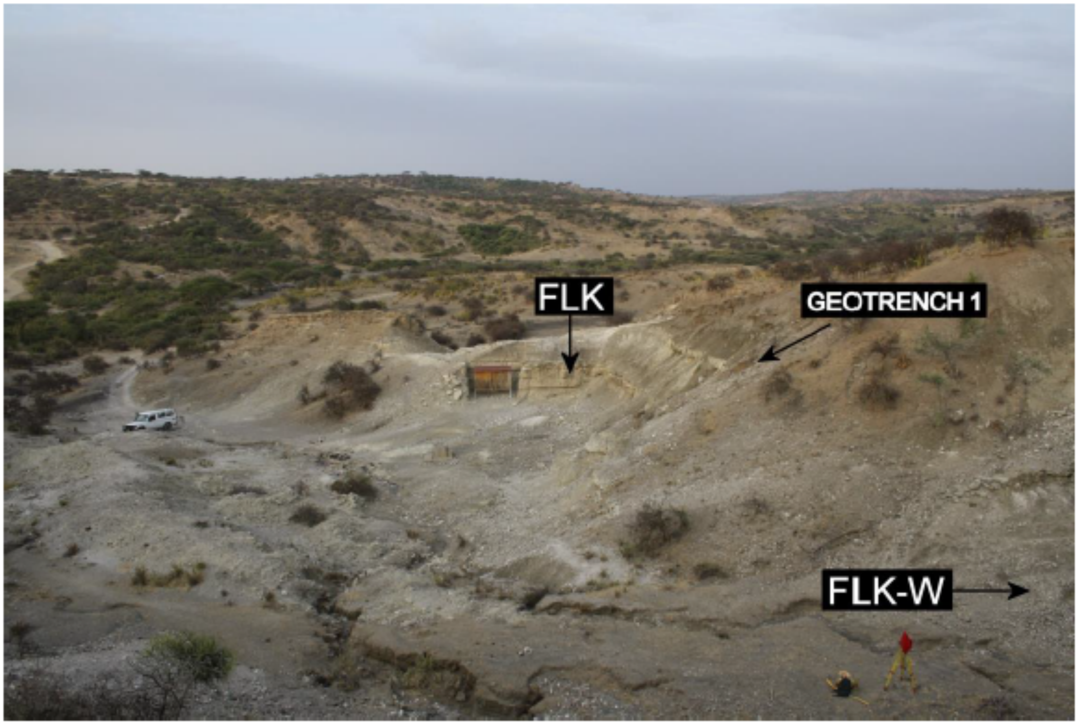

Figure S1.

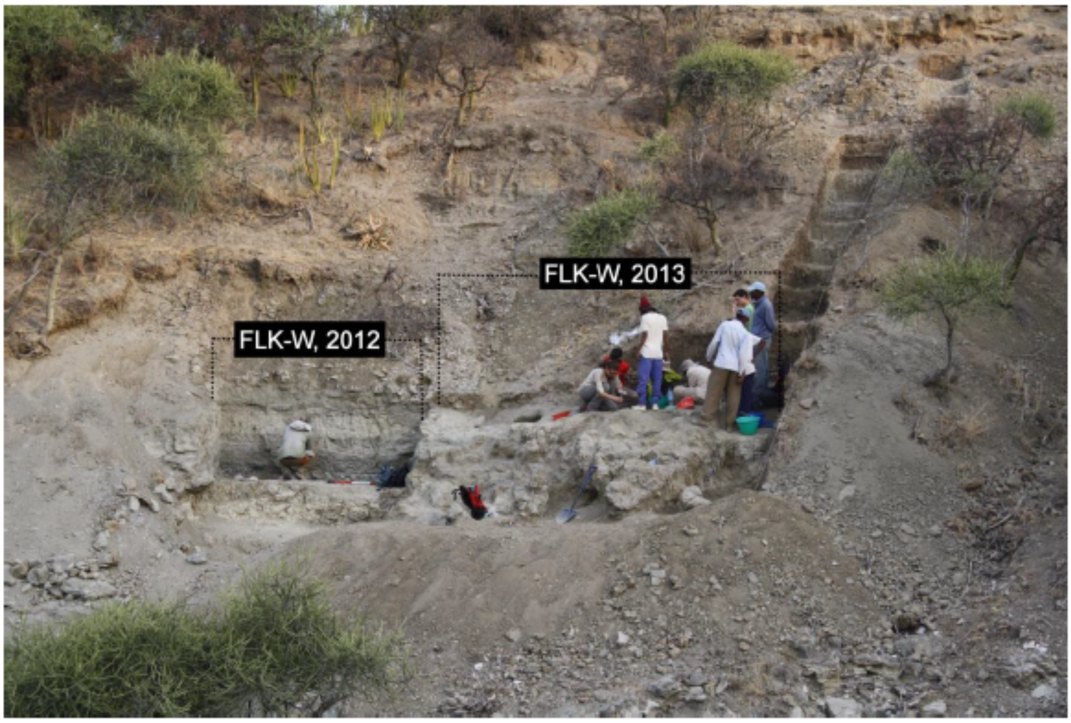

Figure S2

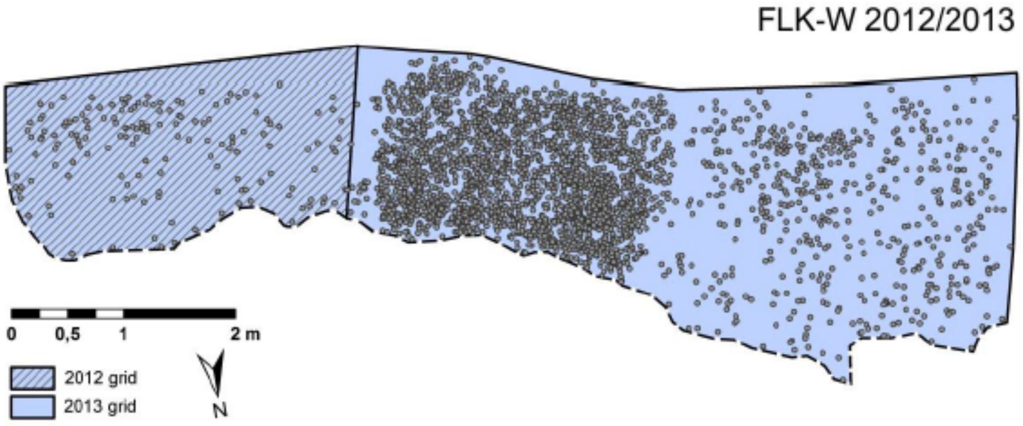

Figure S3.

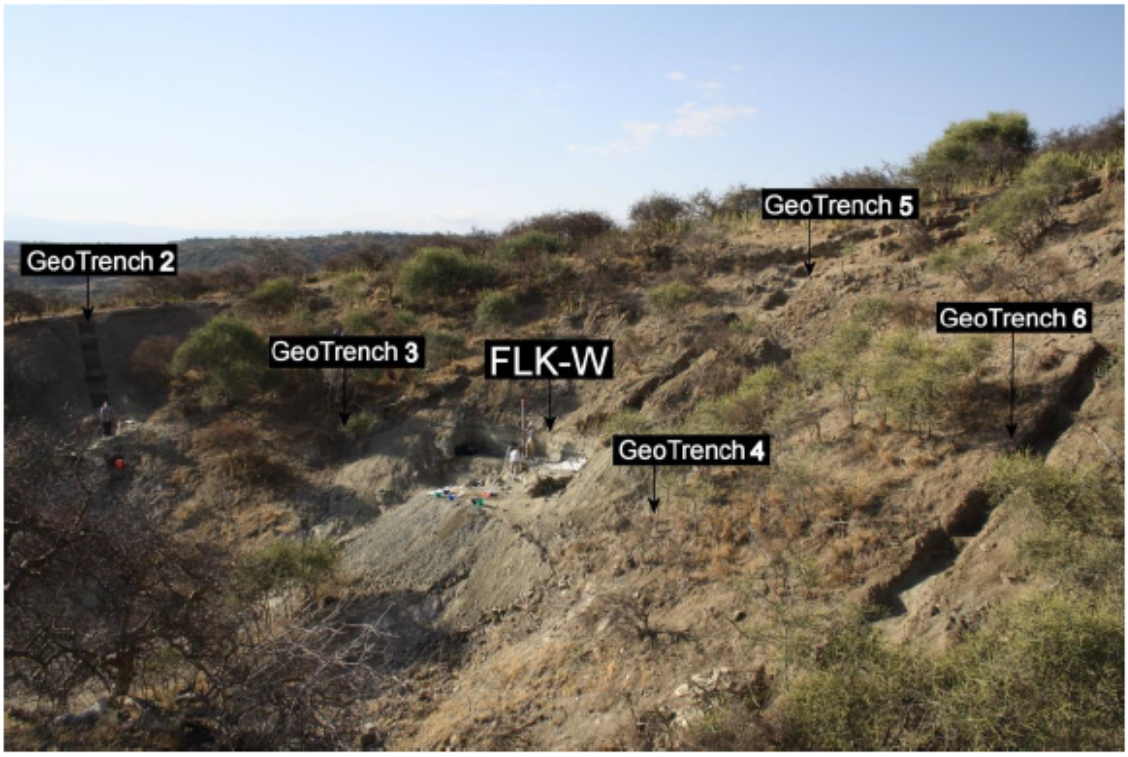

Figure S4.

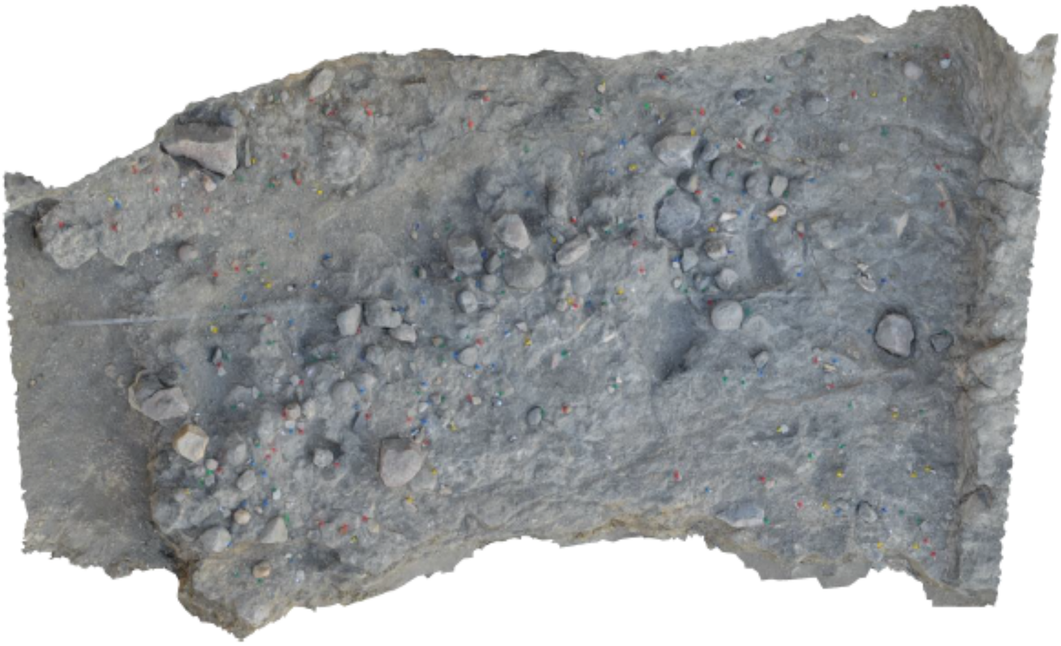

Figure S5.

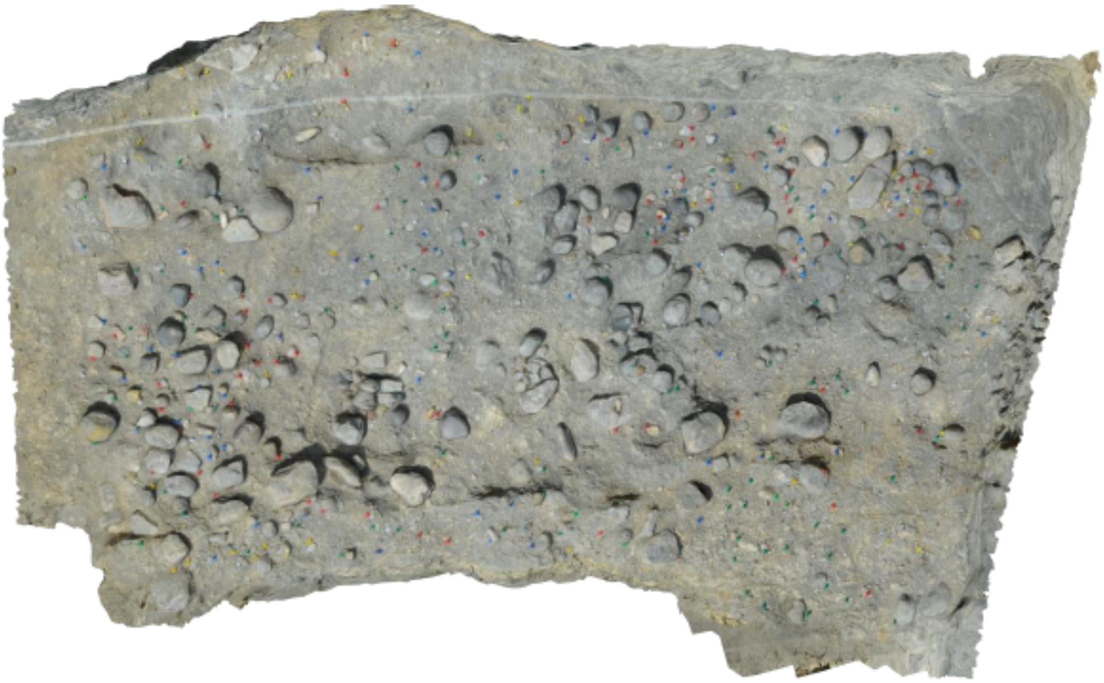

Figure S6.

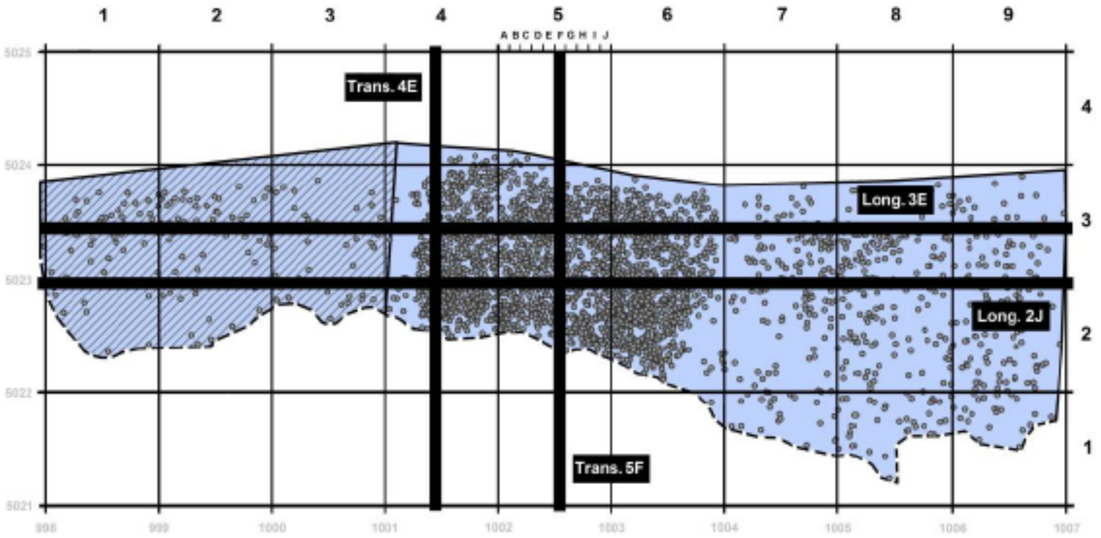

Figure S7.

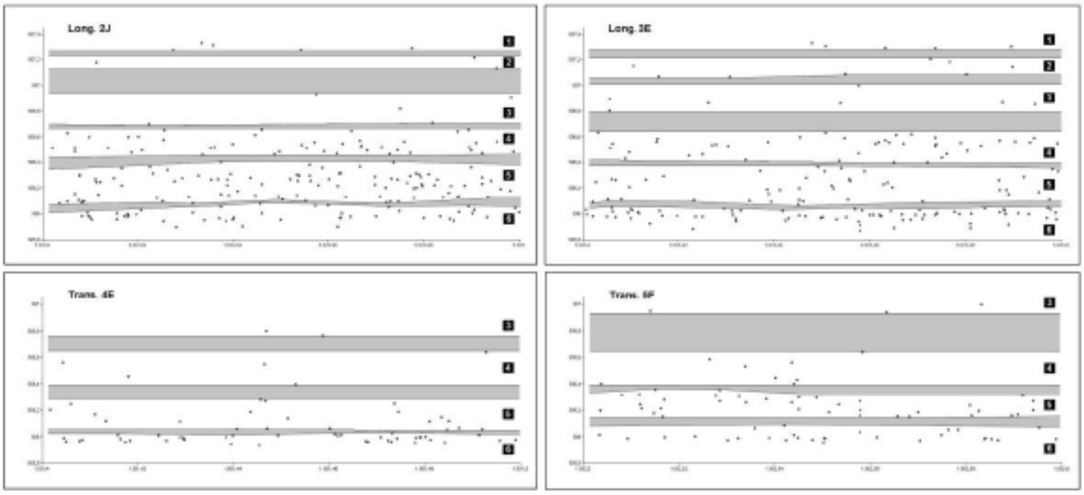

Figure S8.

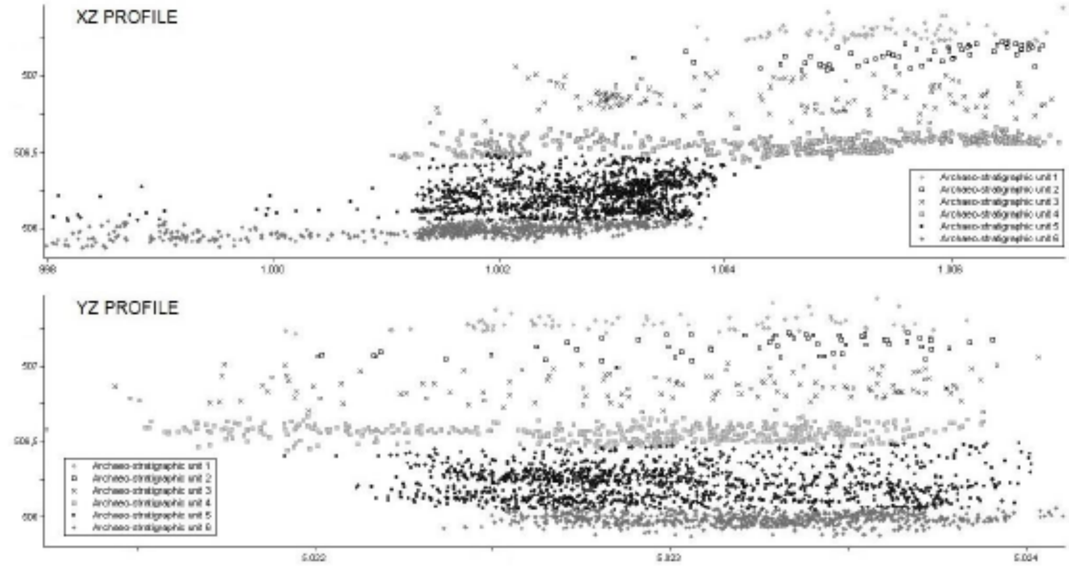

Figure S9.

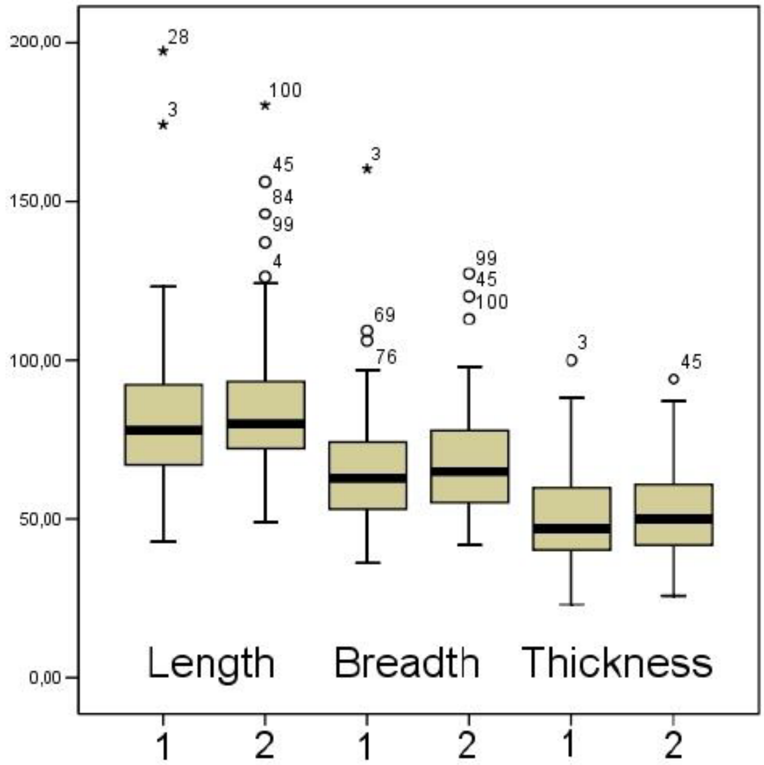

Figure S10.

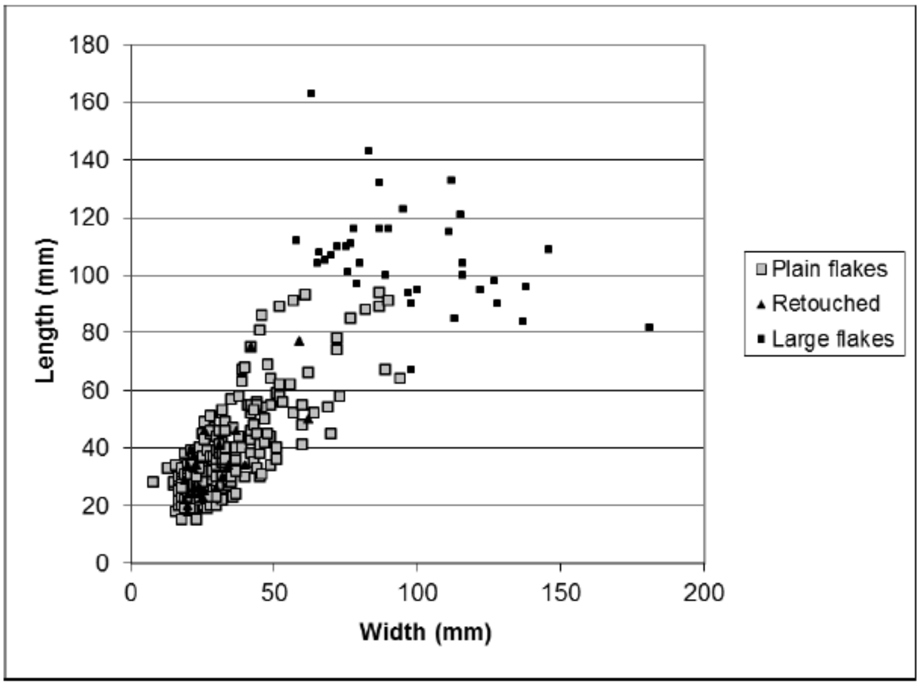

Figure S11.

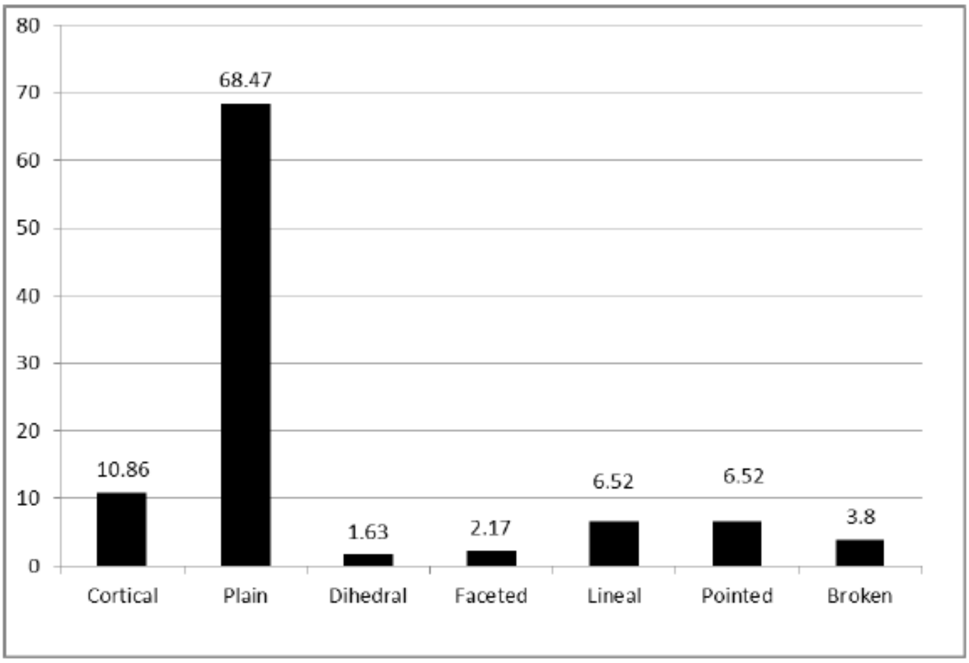

Figure S12.

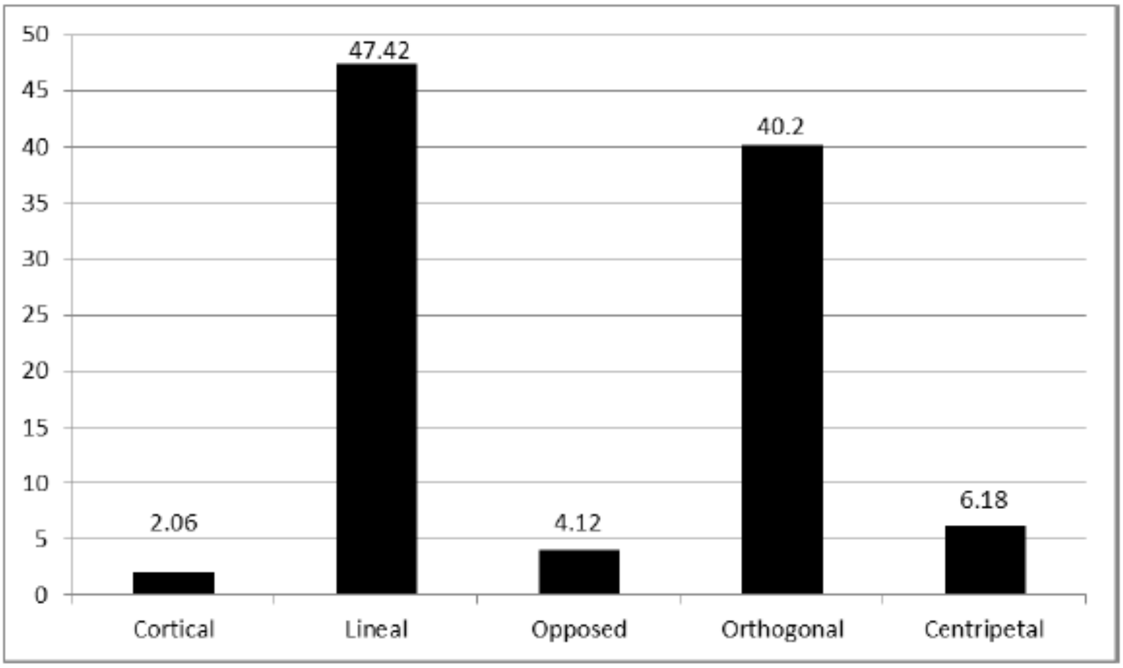

Figure S13.

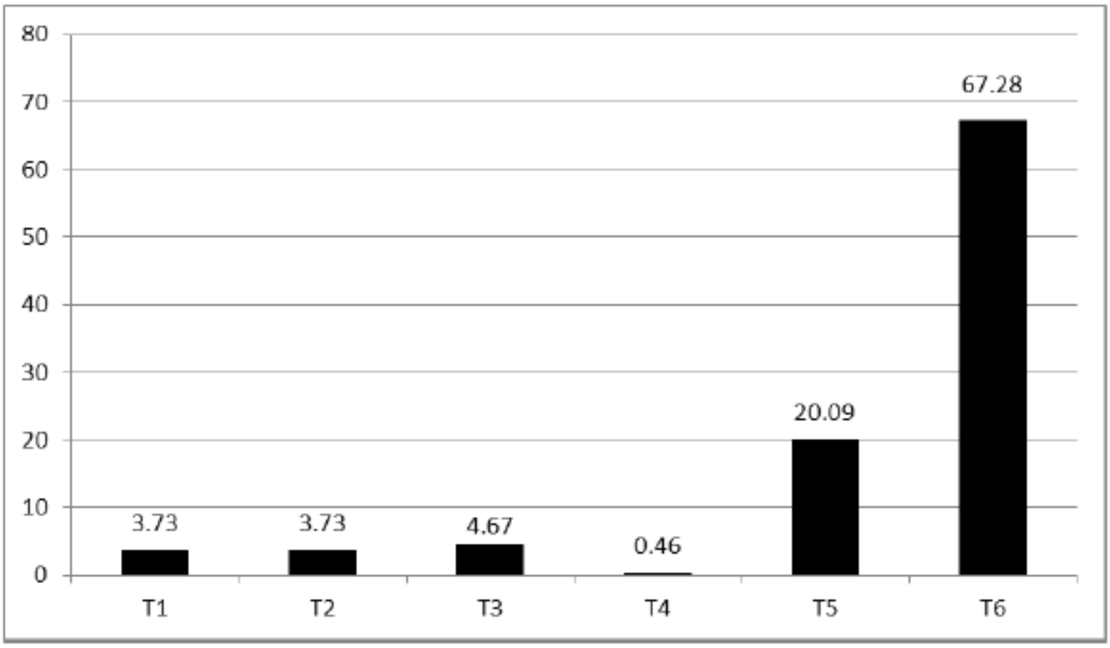

Figure S14.

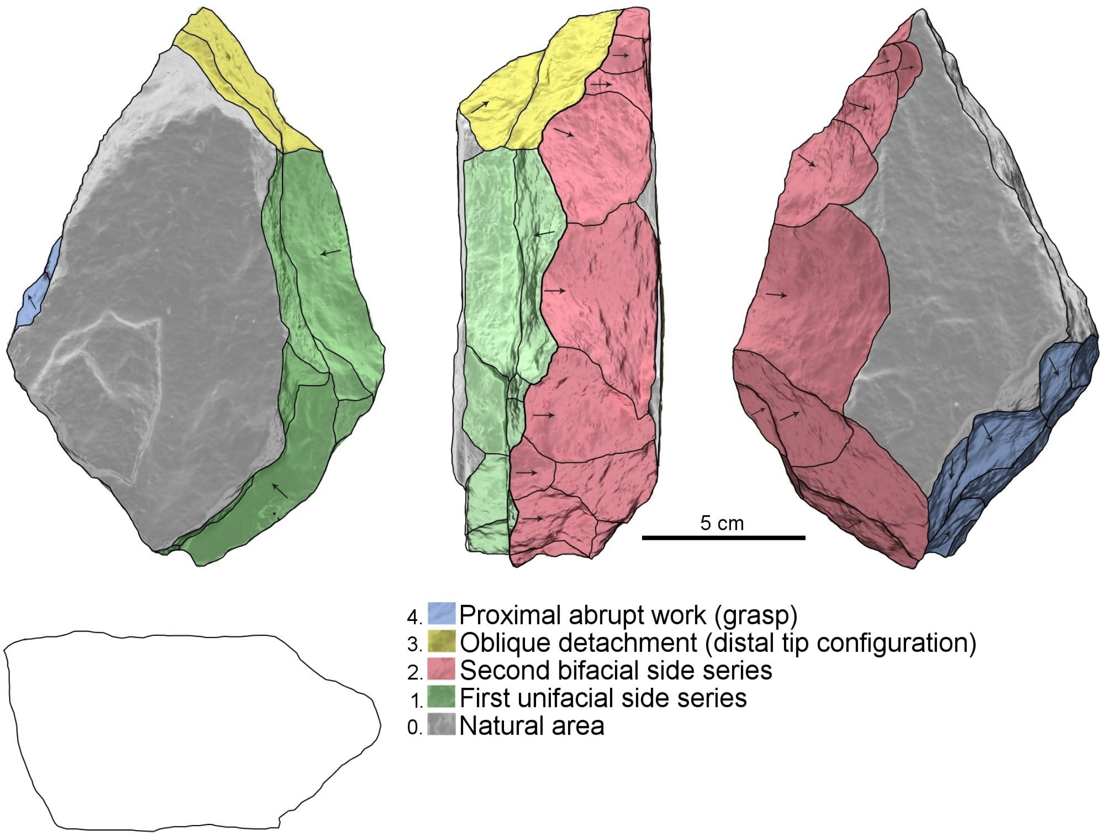

Figure S15.

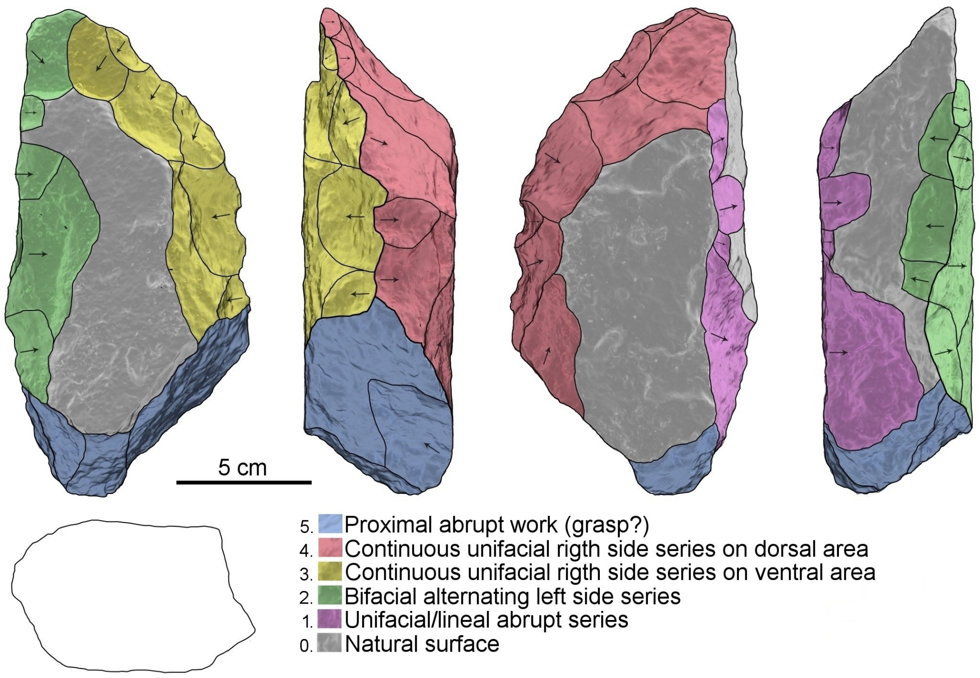

Figure S16.

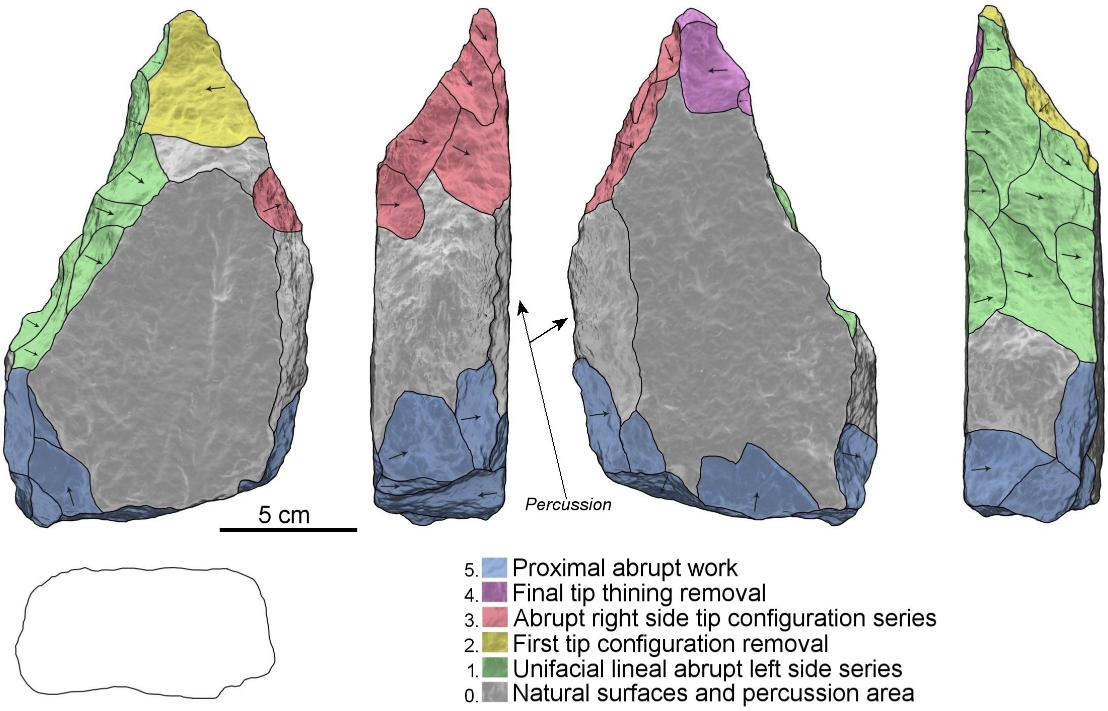

Figure S17.

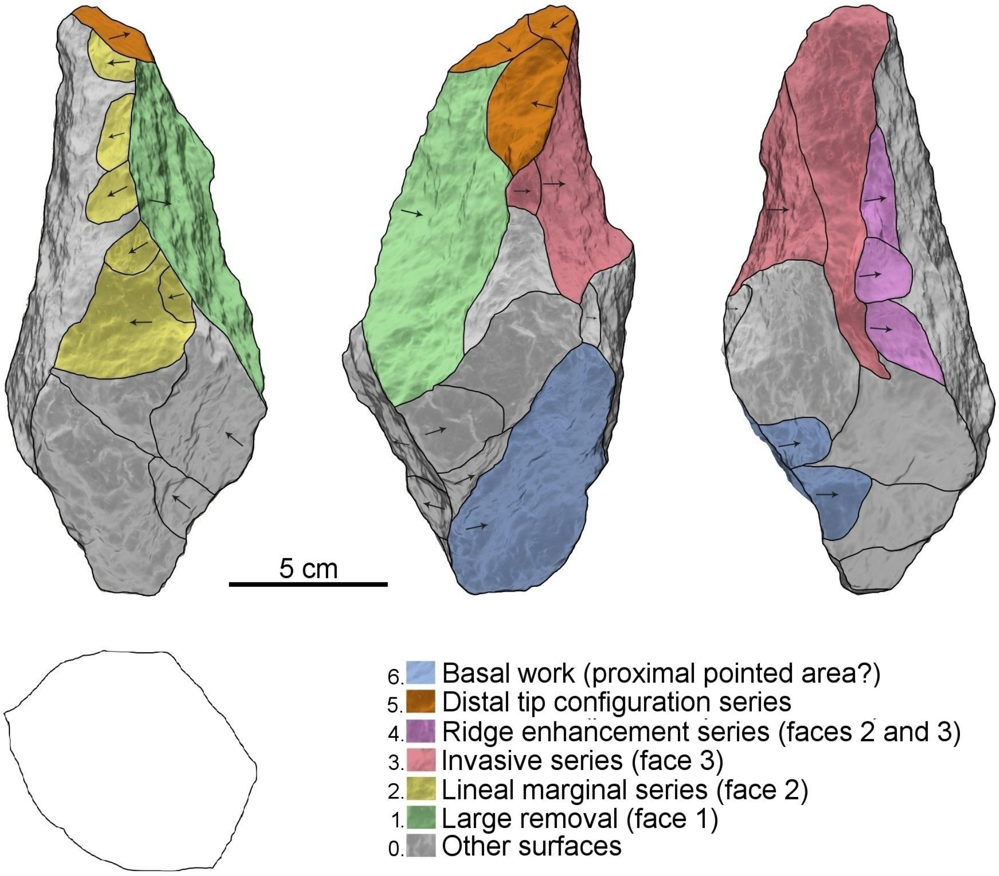

Figure S18.

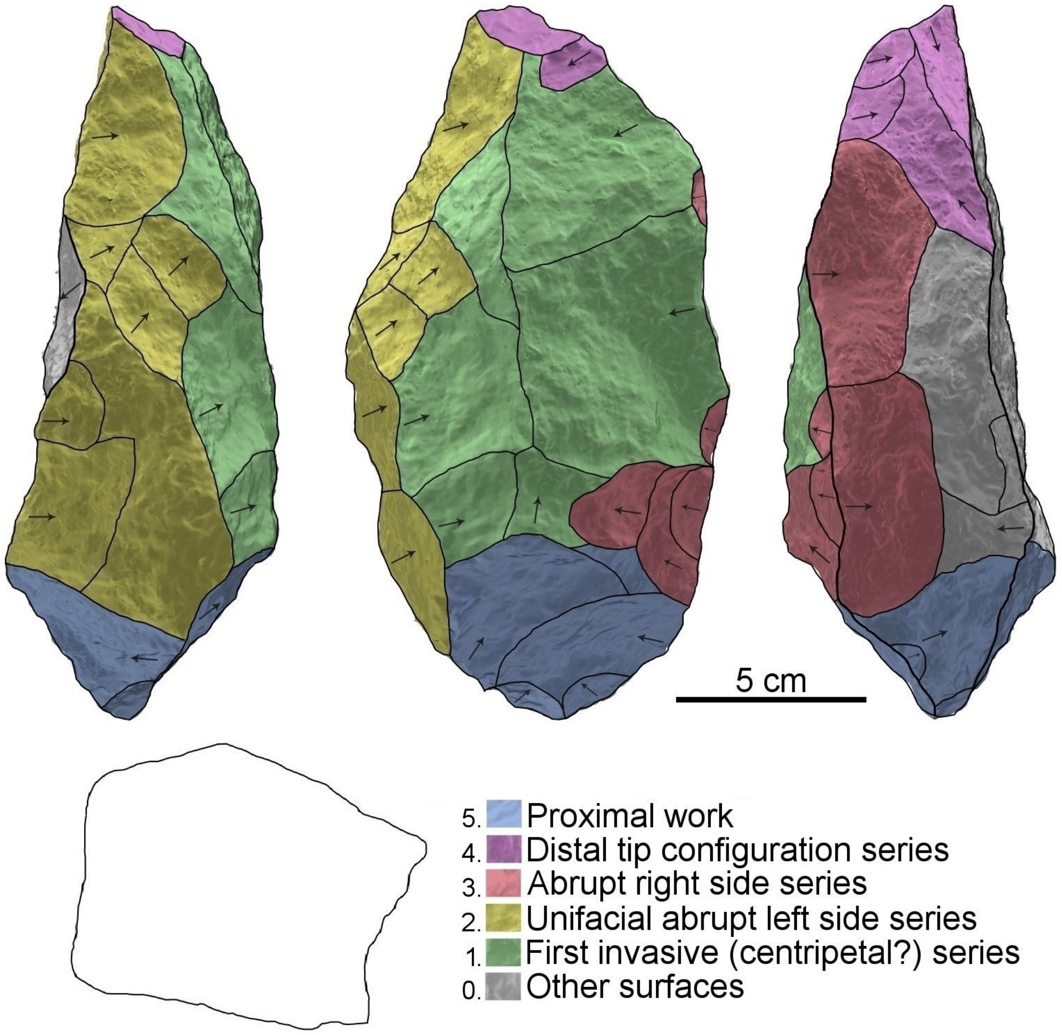
5 cm
5. Proximal work
4. Distal tip configuration series
3. Abrupt right side series
2. Unifacial abrupt left side series
1. First invasive (centripetal?) series
0. Other surfaces

Figure S19.

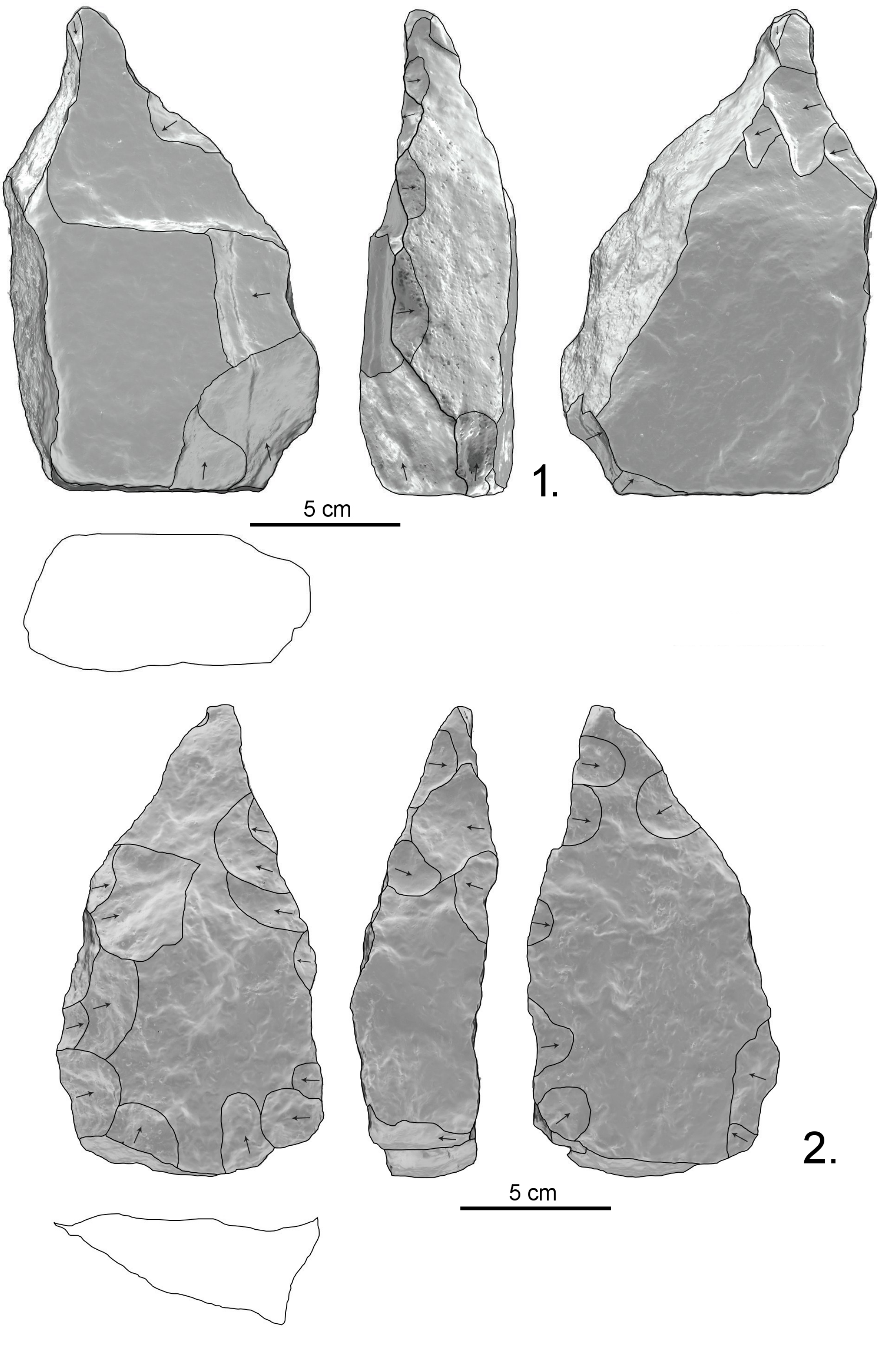

Figure S20.

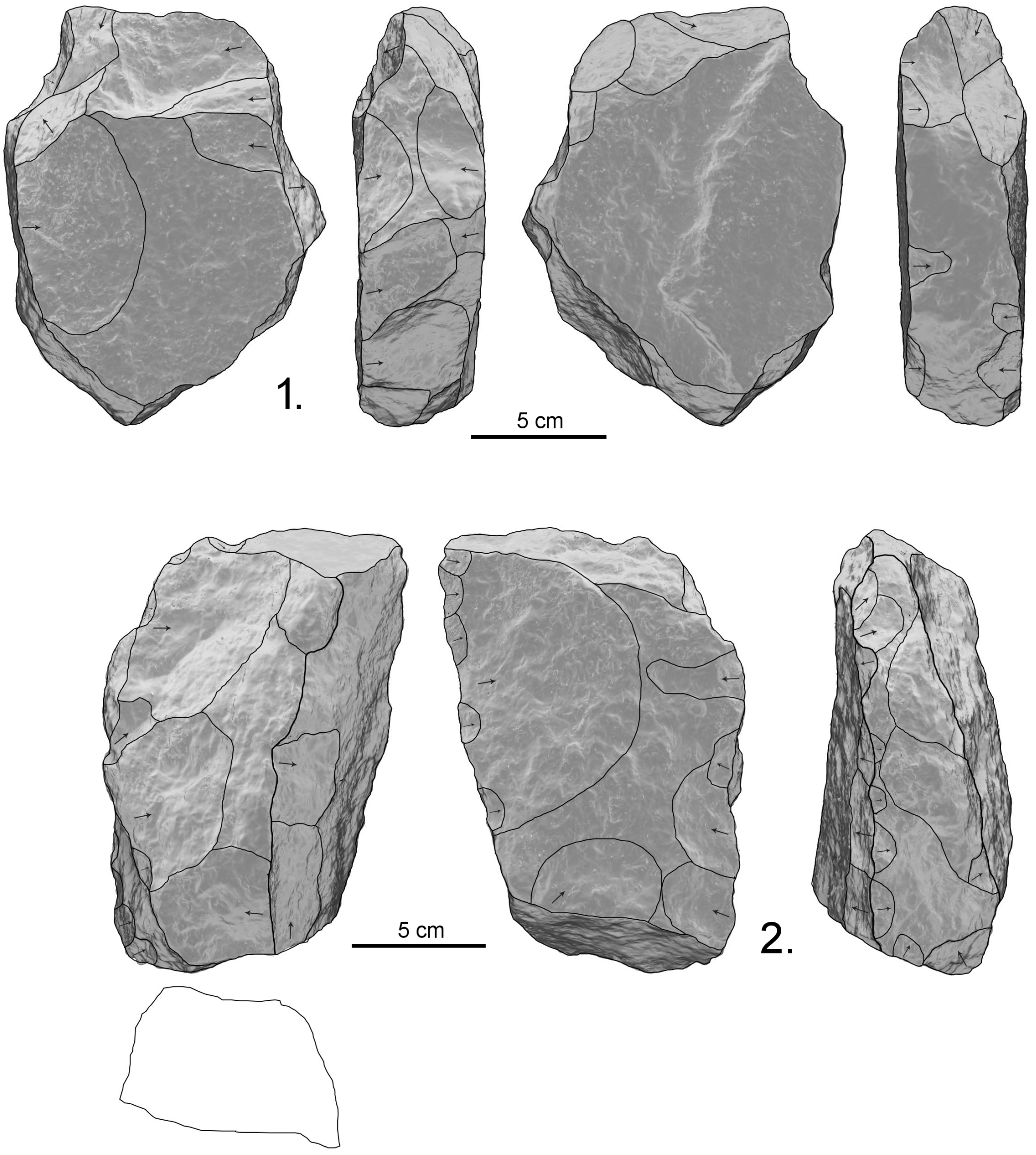

Figure S21.

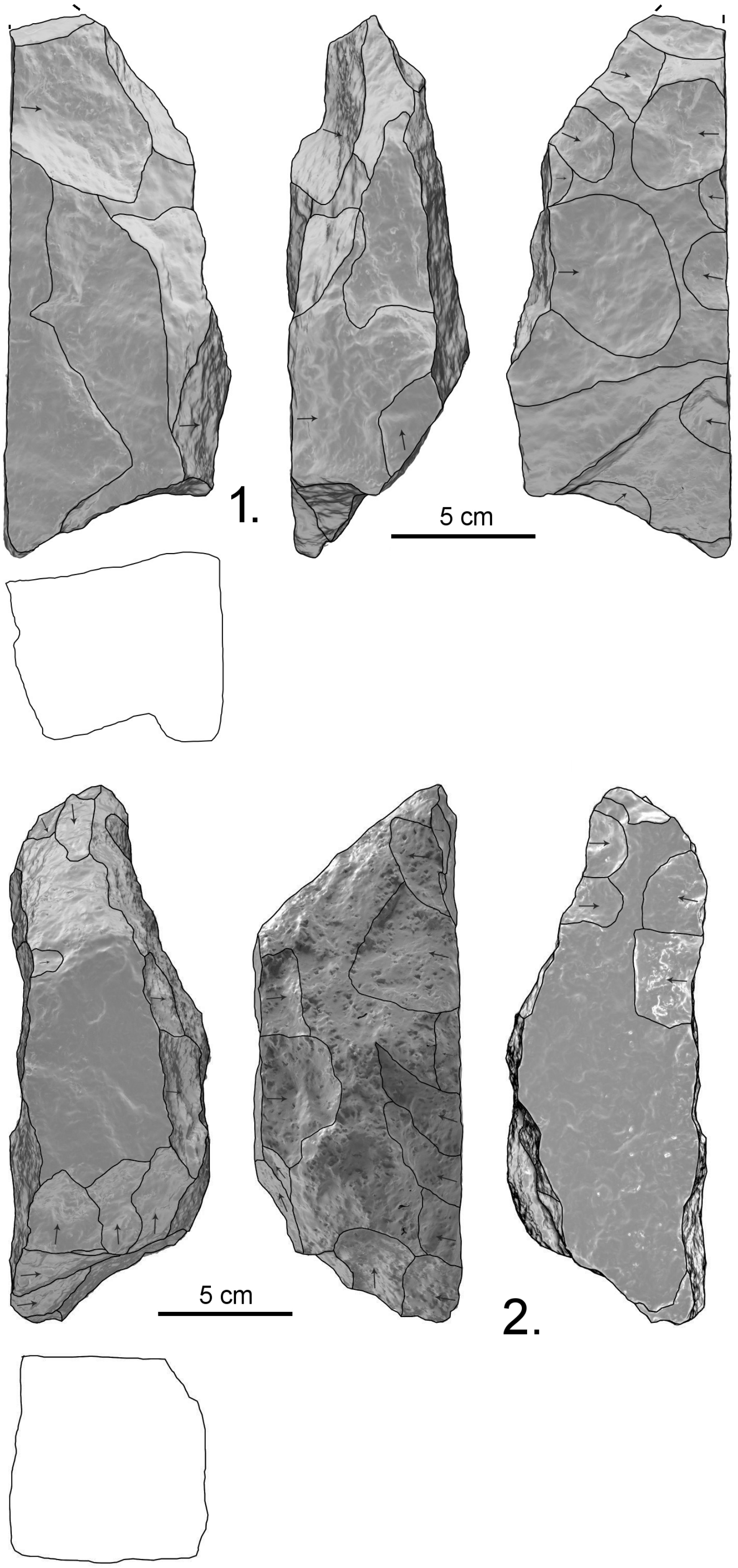

Figure S22.

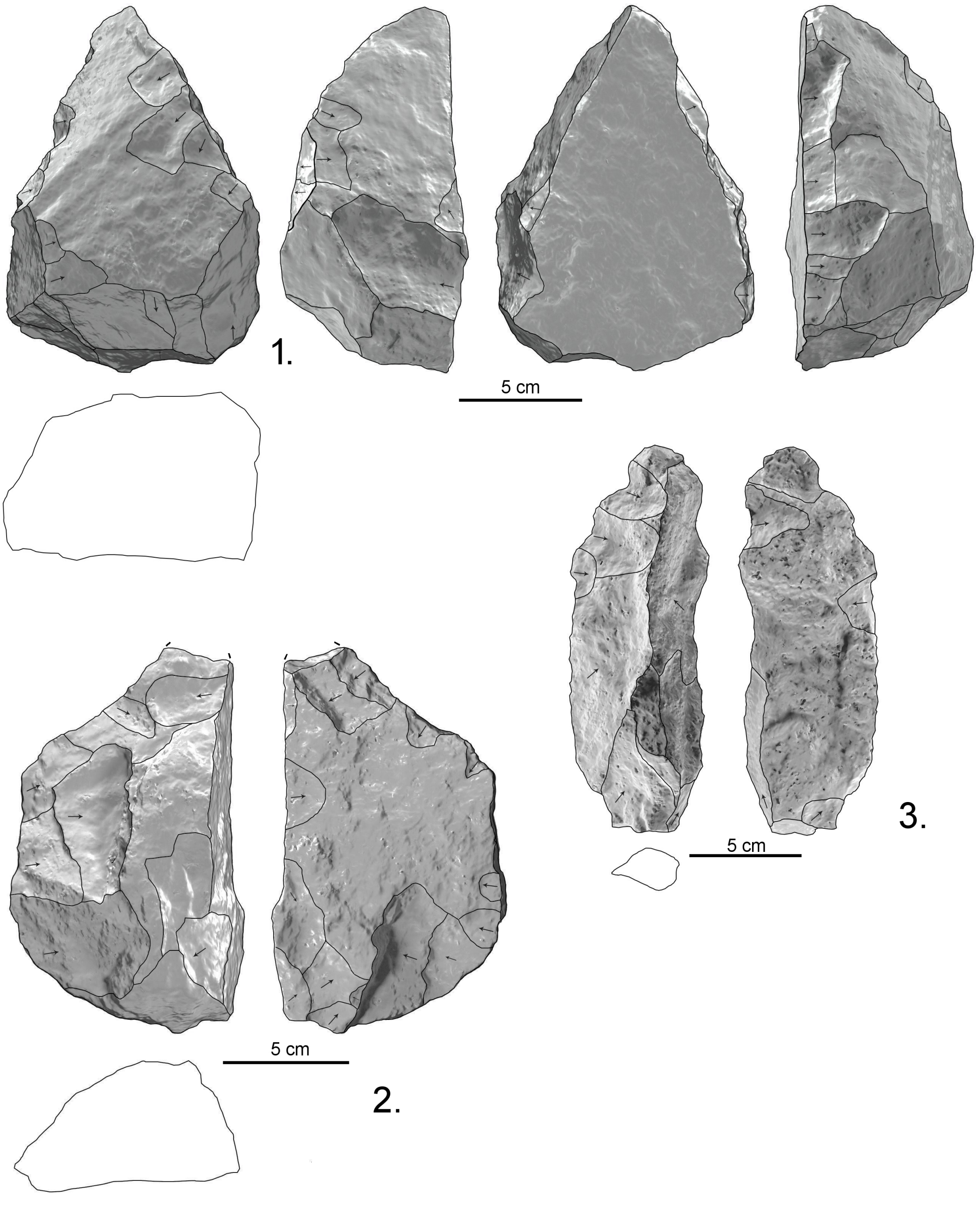

Figure S23.

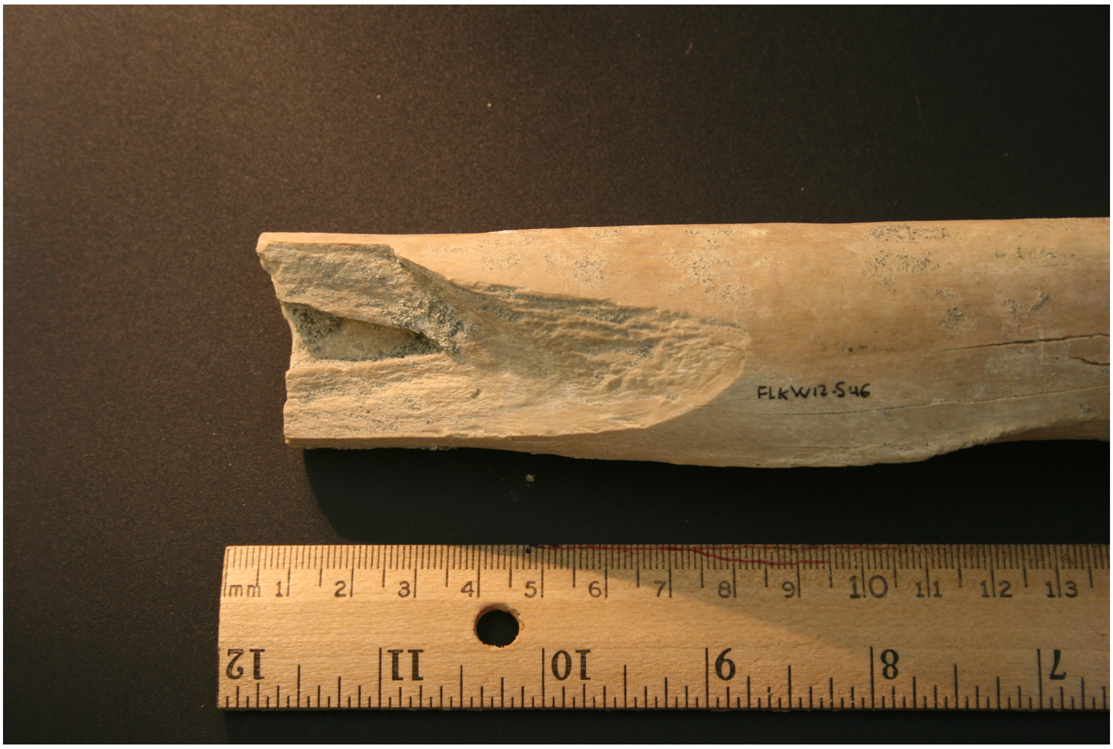

Figure S24.

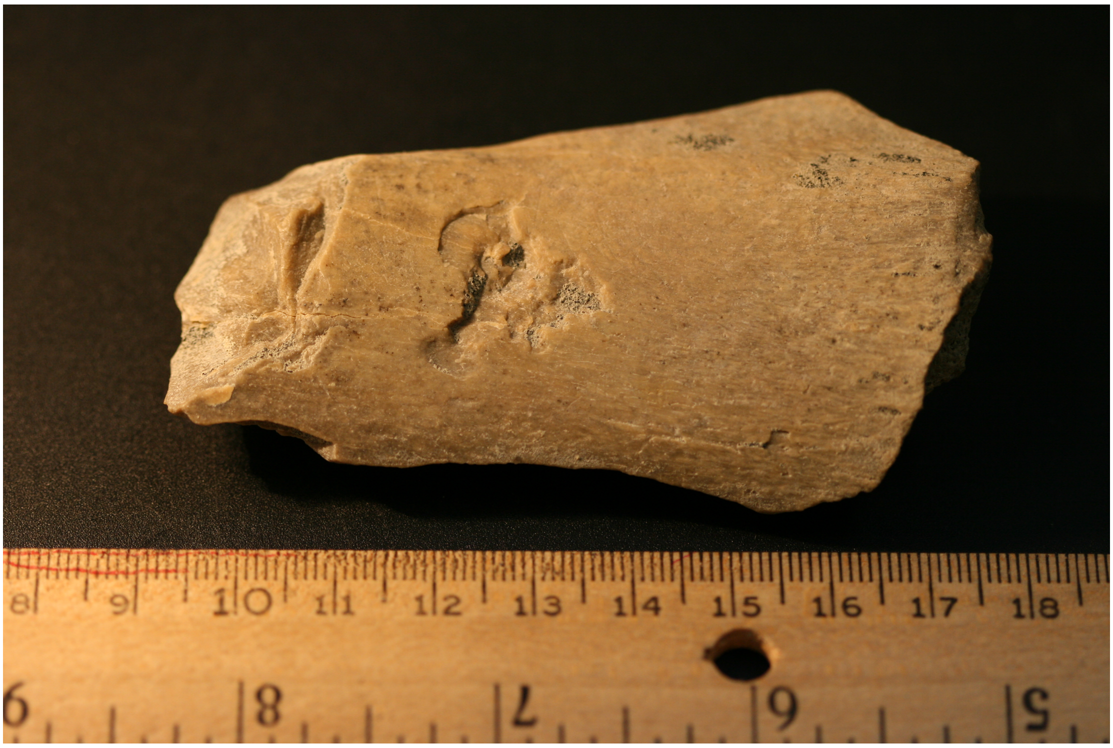

Figure S25.

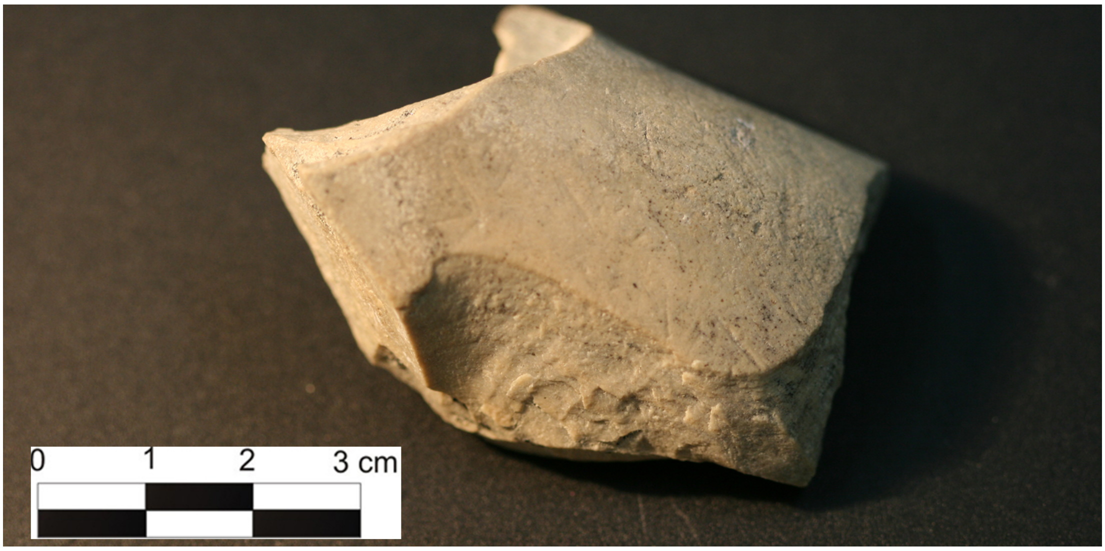

Figure S26.

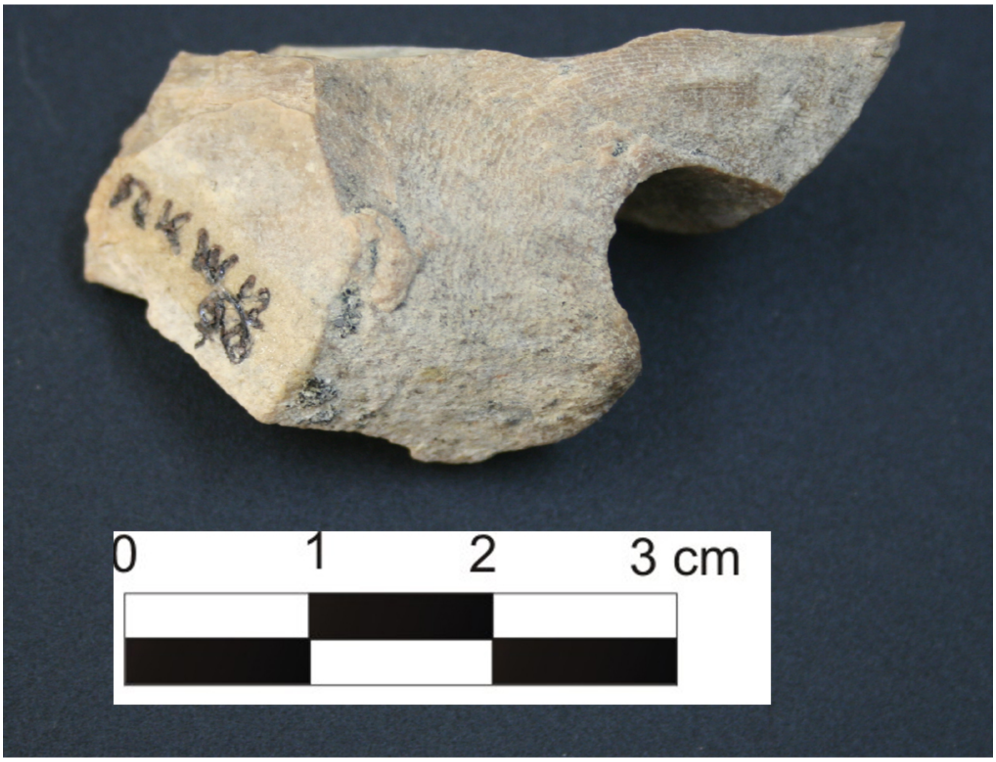

Figure S27.

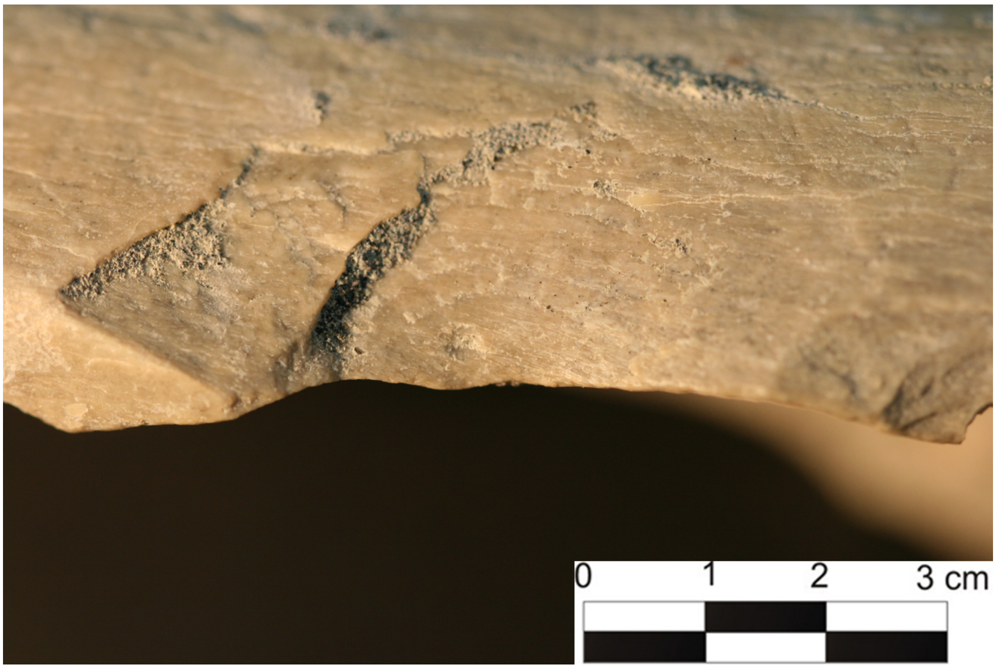

Figure S28.

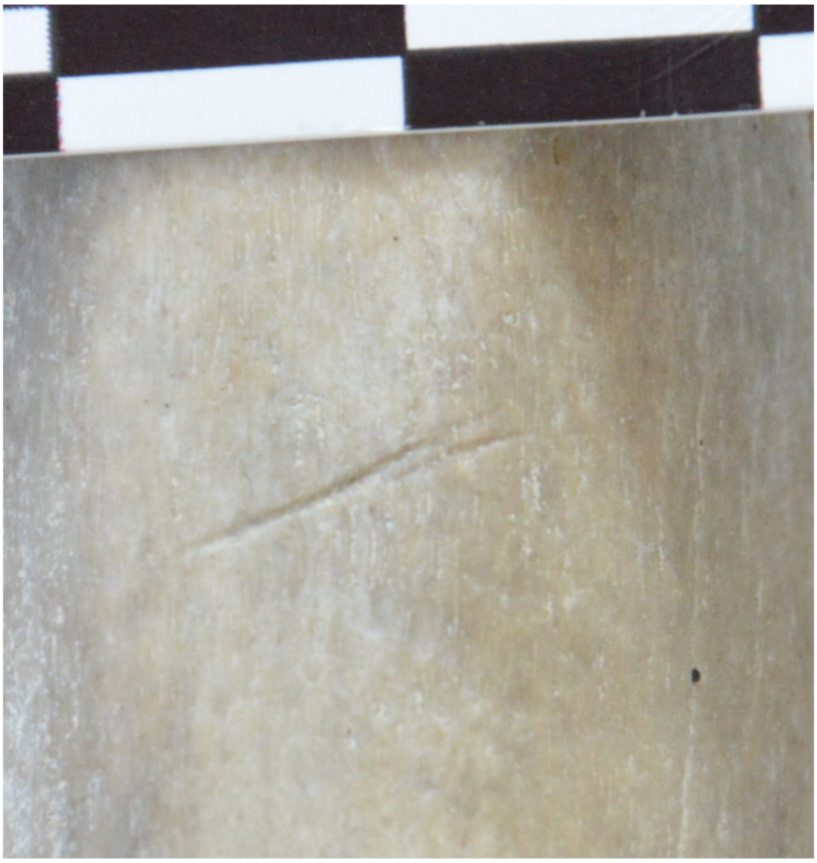
Figure S29.

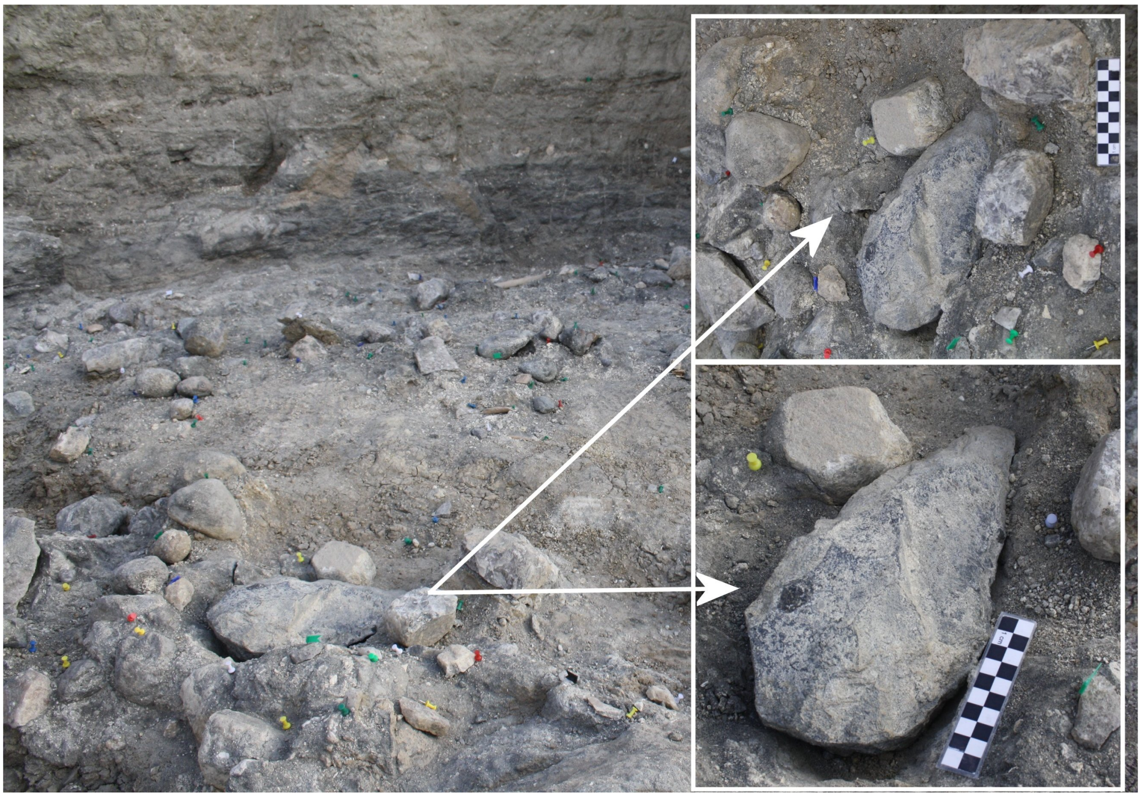

Figure S30.

TABLES

Table S1.

	Basalt	Vesic. Bas.	Phonolite	Gneiss	Quartz	Quartzite	Chert	Total	%
L1	3	-	-	-	22	-	1	26	*1.22*
L2	6	-	1	-	72	1	9	89	*4.19*
L3	31	-	8	-	52	-	6	97	*4.57*
L4	32	2	1	-	220	2	29	286	*13.49*
L5	132	9	12	1	580	10	43	787	*37.12*
L6	100	17	32	3	616	5	62	835	*39.38*
Total	304	28	54	4	1562	18	150	2120	
%	*14.33*	*1.32*	*2.54*	*0.18*	*73.67*	*0.84*	*7.07*		

Table S2.

	R0	R1	R2	R3	Total
L1	9	-	-	-	9
L2	26	1	-	-	27
L3	59	2	-	-	61
L4	91	6	-	1	98
L5	264	9	14	3	290
L6	308	31	21	1	361
Total	757	49	35	5	846
%	89.47	5.79	4.13	0.59	

Table S3.

	Cobbles	Percussion	Fragments	Cores	Detached	Choppers	LCTs	Total	%
L1	2	1	5	1	17	-	-	26	*1.22*
L2	2	4	18	3	62	1	-	89	*4.19*
L3	22	11	10	21	36	-	-	97	*4.57*
L4	14	9	61	15	188	-	-	286	*13.49*
L5	65	38	122	68	495	1	2	787	*37.12*
L6	45	57	142	125	450	3	24	835	*39.38*
Total	150	120	358	213	1248	5	26	2120	
%	7.07	5.66	16.88	10.04	58.86	0.23	1.22		

Table S4.

Level	Anvil	Freehand									
	Bipolar	Test	Unifacial			Bifacial			Multifacial	Large flk.	Exhaust.
			L	O	C	L	O	C			
L1	1	-	-	-	-	-	-	-	-	-	-
L2	1	-	-	-	1	-	-	-	-	-	-
L3	1	4	3	-	-	-	3	1	3	-	2
L4	2	1	1	-	1	1	2	2	3	-	1
L5	8	13	16	-	-	12	4	1	7	3	-
L6	20	16	10	3	2	17	7	3	24	3	10
Total	33	34	30	3	4	30	16	7	37	6	13
%	15.49	15.96	14.08	1.4	1.87	14.09	7.51	3.28	17.37	2.81	6.10

Table S5.

	Perc. flake	Flked indet.	Debris	Flake frag.	Fract. Flake	Whole flake	Retouched	Large Flake	Total	%
L1	1	-	7	-	2	5	2	-	17	*1.36*
L2	1	-	44	2	4	9	2	-	62	*4.96*
L3	1	5	15	2	8	4	1	-	36	*2.88*
L4	-	7	101	29	10	33	7	1	188	*15.06*
L5	3	29	307	22	27	98	8	1	495	*39.66*
L6	7	71	157	35	32	107	7	34	450	*36.05*
Total	13	112	631	90	83	256	27	36	1248	
%	*1.04*	*8.97*	*50.56*	*7.21*	*6.65*	*20.51*	*2.16*	*2.88*		

Table S6.

	Min.	Max.	Mean	Stdard. Dev.
Plain flakes				
Length	15	94	38.39	16.9
Breath	8	95	34.11	16.42
Thickness	3	41	14.79	7.06
Retouched flakes				
Length	18	77	34.57	14,75
Breath	19	62	28.53	11.45
Thickness	5	38	14.11	7.45
Large flakes				
Length	67	163	106.1	17.91
Breath	58	181	97.74	27.94
Thickness	21	64	40.62	10.24

Table S7.

Raw material	No	Dorsal pattern type					Striking platform type				
		Cortical	Lineal	Oposed	Orthogonal	Centripetal	Cortical	Plain	Faceted	Other	
Quartz	203	3	104	6	84	6	19	146	-	38	
Chert	24	2	6	-	14	2	3	13	5	3	
Basalt	21		13	3	5	-	2	14	3	2	
Quartzite	5	-	2	-	1	2	1	3	1	-	
Phonolite	3	-	1	-	1	1	-	2	-	1	

Table S8.

Morpho-functional group	n	Type	Raw material			Blank			Retouch		
			Q	B	P	LF	TP	C	U	B	T
Pointed	n=11	Distal points/tips	9	1	1	4	5	2	2	3	6
Side edge+pointed	n=7	2 convergent edges+distal tip	3	-	-	1	2	-	3	-	-
		1 side edge+distal tip	4	-	-	1	3	-	3	1	-
Cutting edges	n=4	Distal retouched edge	3	1	-	3	1	-	-	4	-
Cleavers	n=2	Crude cleavers	2	-	-	2	-	-	2	-	-
Knives	n=2	Knives	2	-	-	-	2	-	1	1	-

Table S9.

Raw material	Length	Breadth	Thickness	Weight
Quartz	98	70	43	479
Quartz	99	78	36	480
Basalt	108	100	60	774
Quartz	117	95	40	526
Quartz	122	74	43	441
Quartz	125	76	55	617
Quartz	135	84	49	586
Quartz	144	93	46	773
Quartz	144	65	57	673
Quartz	147	90	43	778
Quartz	150	104	1455	1010
Phonolite	152	53	36	189
Quartz	156	119	46	1324
Quartz	159	82	41	600
Quartz	164	106	69	1619
Quartz	167	103	51	1145
Quartz	180	72	70	865
Quartz	180	120	63	1399
Quartz	182	86	56	1180
Quartz	185	96	46	1269
Quartz	185	95	60	1280
Quartz	188	88	77	1107
Quartz	190	55	76	1072
Quartz	216	76	76	1755
Basalt	310	140	83	3660

Table S10.

	Level 5		Level 6	
	NR	MNI	NR	MNI
alcelaphini 2	2	1		
alcelaphini 3a	5	2	4	1
alcelaphini 3b	1	1	1	1
Megalotragus sp.			3	1
Hippotragus gigas			1	1
Giraffidae	1	1	1	1
Pelorovis oldowayensis	1	1		
Kolpochoerus sp.	2	1	3	1
Metridiochoerus sp.	2	1	6	2
Suidae	2		1	
Equus oldowayensis	4	2	7	1
Hipparion cornelianum			8	3
Indet size 1	4		4	
Indet size 2	26		19	
Indet size 3	38		29	
Indet size 3a	29		29	
Indet size 3b	13		37	
Indet size 4	19		29	
Indet size 5	5		9	
Indet size 6			1	
indet	69		93	
Total	221	8	282	12

Table S11.

		small	medium	large
Level 5				
	CM	0/22(0)	1/37(2.7)	2/6(33.3)
	PM	0/22(0)	2/37(5.4)	3/6(50)
	TM	4/22(18)	4/37(10.8)	1/6(16.6)
Level 6				
	CM	0/13(0)	1/34(2.9)	0/13(0)
	PM	0/13(0)	5/34(14.7)	3/13(23)
	TM	2/13(15.3)	3/34(8.8)	0/13(0)

$^{40}Ar/^{39}Ar$ supplementary information

Methods & data summary

Samples were disaggregated gently using a mortar and pestle. Sanidine was handpicked under a binocular microscope using the methods of Hyneck et al. (2010) to ensure a pure separate. After leaching in dilute HF and rinsing in de-ionised water and methanol, the grains were parcelled into Cu packets and positioned within an Al holder for irradiation. International standard Alder Creek Tuff sanidine (ACs, 1.193 ± 0.001 Ma; Nomade et al. 2005), a secondary standard referenced against the Fish Canyon age (FCs) (28.02 ± 0.16 Ma) of Renne et al. (1998), was loaded adjacent to the samples of unknown age. Samples were irradiated for 180 minutes in the Cd-lined CLICIT facility of the OSU TRIGA reactor. ACs grains (*n* = 40) were analysed by total fusion with a focused CO_2 laser. The J-parameter was determined to a precision of c. 0.1 %.

Single grains (100-200 μm) of unknowns (note crystals were exceptionally small) were loaded into a Cu planchette in an ultra-high vacuum laser cell with a doubly pumped ZnSe window. Using a CO_2 laser, the sanidine crystals were degassed at low temperature and subsequently fused. Radiogenic ^{40}Ar ($^{40}Ar^*$) yields were thus improved from 50-60% to typically greater than 80%; no ^{39}Ar was liberated. All gas fractions were subjected to 180 s of purification with two SAES GP50 getters (one at room temperature the other at 450 ˚C) and a cold finger maintained at -95.5 ˚C using a mixture of dry ice ($CO_{2[s]}$) and acetone. Argon isotope ratios (i.e., ion beam intensities) were measured using a MAP 215-50 mass spectrometer in peak jumping mode. The mass spectrometer has a measured sensitivity of 1.13×10^{-13} moles/volt. Both the extraction and cleanup processes were automated, as were the mass spectrometer peak jumping routines and data acquisition. Backgrounds were measured after every two analyses of unknowns and average backgrounds ± standard deviation from the entire run sequence were used to correct raw isotope measurements of unknowns. Mass discrimination was monitored by analysis of air pipettes after every five analyses. Backgrounds and discrimination (D) are reported in the excel spreadsheet containing the raw $^{40}Ar/^{39}Ar$ data. The Ar isotope data were corrected for backgrounds, mass discrimination, and reactor-produced nuclides (all reported in supplementary Ar/Ar excel spreadsheet) and processed using standard data reduction protocols. The decay constants of Steiger & Jäger (1977) and atmospheric argon ratios of Lee et al. (2006), the latter independently verified by Mark et al. (2012), were employed.

The BGC software *MassSpec* was used for data regression. Data are displayed on ideograms and isotope correlation plots (inverse isochron plots). The standard error on the mean was determined for all samples that displayed a Gaussian (normal) distribution with a MSWD < 1. Data were not rejected on the basis of $\%^{40}Ar^*$. All data form single crystal age populations. Data are reported as X ± Y where Y is the full external precision.

The Ar/Ar method is a relative dating technique with all ages referenced back to a standard of known age. The ACs age of Nomade et al. (2005) is determined relative to FCs with an age of 28.02 ± 0.16 Ma (Renne et al., 1998). More recently there have been a series of updated ages suggested for FCs (Kuiper et al., 2008; Renne et al., 2010, 2011; Rivera et al., 2011). These calibrations have reduced systematic uncertainties in

the Ar/Ar system from *c*. 2.5 to < 0.25 %. We have recalculated our Ar/Ar ages using the optimisation model of Renne et al. (2011). Ar/Ar age uncertainties include full error propagation and these are what are reported in the main text. All raw Ar/Ar data can be located in the accompanying excel spreadsheet (Ar/Ar_data).

FLKWA Ideogram & Inverse isochron

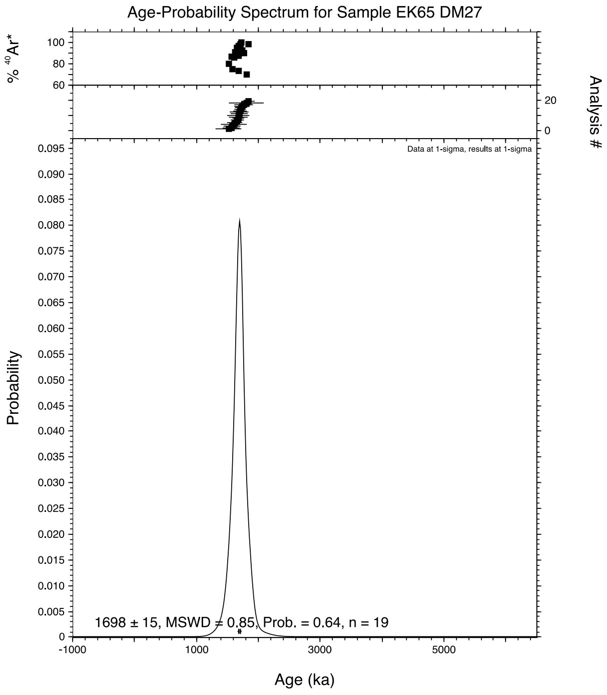

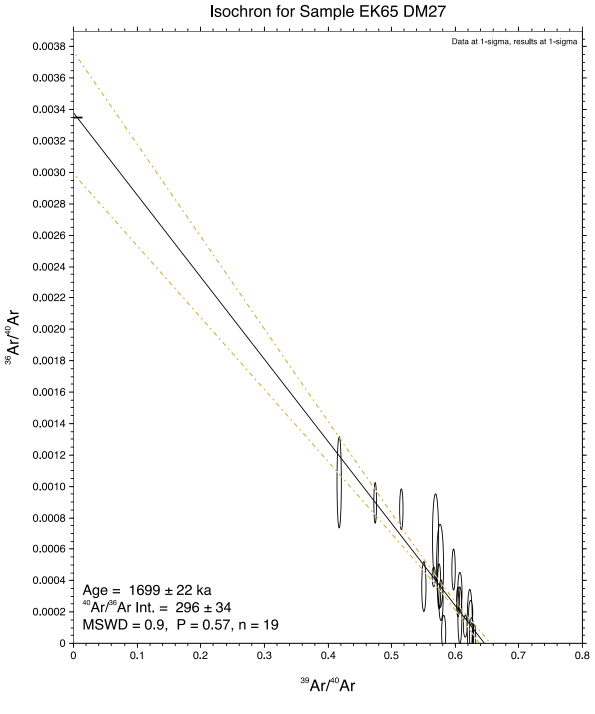

FLKWB Ideogram & Inverse isochron

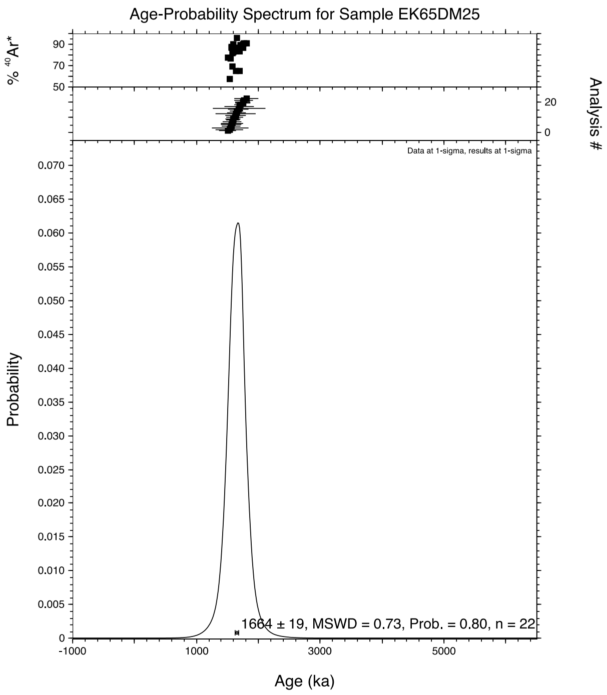
Age-Probability Spectrum for Sample EK65DM25
% ^{40}Ar*
Analysis #
Data at 1-sigma, results at 1-sigma
Probability
1664 ± 19, MSWD = 0.73, Prob. = 0.80, n = 22
Age (ka)

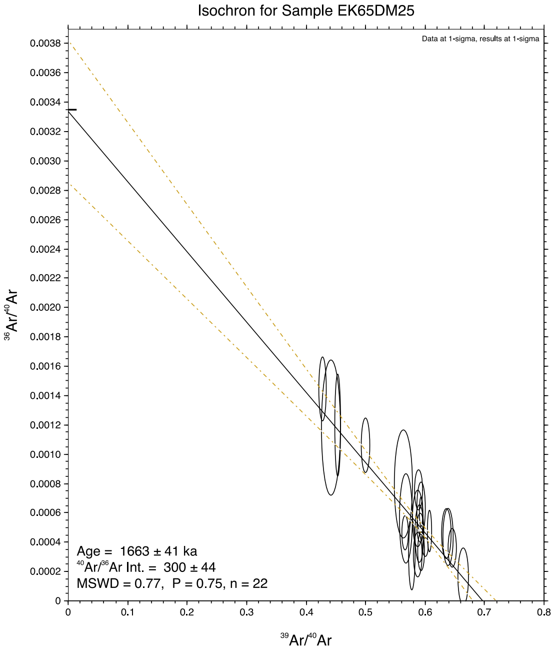
Isochron for Sample EK65DM25
Data at 1-sigma, results at 1-sigma
^{36}Ar/^{40}Ar
Age = 1663 ± 41 ka
^{40}Ar/^{36}Ar Int. = 300 ± 44
MSWD = 0.77, P = 0.75, n = 22
^{39}Ar/^{40}Ar

Ar/Ar results are summarised in the table below and ages are calculated using two different sets of standard ages and decay constants.

Sample	n	MSWD	Steiger & Jager 1977, Renne et al., 1998 (Ma ±1s analytical)	Renne et al., 2011 (Ma ± 1s full external precision)
FLKW-A	22/22	0.9	1.681 ± 0.015	1.698 ± 0.015
FLKW-B	19/19	0.7	1.648 ± 0.019	1.664 ± 0.019

Data calculated relative to the optimization model of Renne et al. (2011) are reported throughout the main text.

References

Hynek, S.A., Brown, F.H., and Fernandez, D.P., 2010. A rapid method for hand picking potassium-rich feldspar from silicic tephra. Quaternary Geochronology, 6, 285-288.

Kuiper, K.F., Deino A., Hilgen, F.J., Krijgsman W., Renne, P.W., Wijbrans J.R., 2008. Synchronizing Rock Clocks of Earth History. Science 25, 320:500-504.

Lee, J.-L., Marti, K., Severinghaus, J.P., Kawamura, K.. Yoo, H.-S., Lee, J.B., Kim, J.S. 2006. A redetermination of the isotopic abundances of atmospheric Ar. Geochimica et Cosmochicima Acta 70, 4507–4512.

Mark, D.F., Stuart, F.M., de Podesta, M. (*in press*) New high-precision measurements of the isotopic composition of atmospheric argon. Geochimica et Cosmochimica Acta, doi:10.1016/j.gca.2011.09.042

Nomade, S., Renne, P. R., Vogel, N., Deino, A. L., Sharp, W. D., Becker, T. A., Jaouni, A. R., Mundil, R., 2005. Alder Creek sanidine (ACs-2): a Quaternary $^{40}Ar/^{39}Ar$ dating standard tied to the Cobb Mountain geomagnetic event. Chemical Geology, 218, 315-338.

Renne, P. R., Mundil, R., Balco, G., Min, K., Ludwig, K. R., 2010. Joint determination of ^{40}K decay constants and $^{40}Ar^*/^{40}K$ for the Fish Canyon sanidine standard, and improved accuracy for $^{40}Ar/^{39}Ar$ geochronology. Geochimica et Cosmochimica Acta, 74, 5349–5367

Renne P. R., Swisher C. C., Deino A. L., Karner D. B., Owens T. L., DePaolo D. J., 1998. Intercalibration of standards, absolute ages and uncertainties in $^{40}Ar/^{39}Ar$ dating. Chemical Geology, 145, 117– 152.

Renne P.R., Balco G., Ludwig, K.R., Mundil, R., Min, K., 2011. Response to the comment by W.H. Schwarz et al. on "Joint determination of ^{40}K decay constants and ^{40}Ar*/^{40}K for the Fish Canyon sanidine standard, and improved accuracy for ^{40}Ar/^{39}Ar geochronology" by P.R. Renne et al., 2010. Geochimica et Cosmochimica Acta 75, 5097-5100.

Rivera, T.A., Storey, M., Kuiper, K., Pälike, H., 2011. Towards an Integrated Geomagnetic Polarity Reversal Timescale for the Pleistocene. AGU Fall meeting, San Francisco, USA.

Steiger, R.H., Jäger, E., 1977. Subcommission on geochronology: convention on the use of decay constants in geo- and cosmochronology. Earth and Planetary Science Letters, 36, 359-362.

Figure captions

Figure S1. View of FLK and related location of FLK West site and GeoGrench 1. Photograph taken by F. Diez-Martín.

Figure S2. View of the 2012 test pit and the 2013 archaeological excavation at FLK West. Photograph taken by F. Diez-Martín.

Figure S3. Excavated areas opened in 2012 and 2013 in FLK West. Photograph taken by F. Diez-Martín.

Figure S4. Panoramic view of FLK West and GeoTrenches 2-6. Photograph taken by F. Diez-Martín.

Figure S5. Orthofotography of a horizontal plane in Level 5 at FLK West, showing the archaeological associations. Image made by E. Organista

Figure S6. Orthofotography of a horizontal plane in Level 6 at FLK West, showing the archaeological associations. Image made by E. Organista.

Figure S7. Grid used for the archaeo-stratigraphic analysis, showing the location of the longitudinal (2J and 3E) and transversal (4E and 5F) projections reproduced in figure 8. Drawing made by F. Diez-Martín.

Figure S8. Examples of detailed longitudinal (2J and 3E) and transversal (4E and 5F) archaeo-stratigraphic projections. The grey bands are the depositional disruptions observed between the different archaeo-units. Drawing made by F. Diez-Martín.

Figure S9. XZ and YZ vertical projections of the archaeological materials unearthed from FLK West sorted by archaeo-stratigraphic units (1-6 from top to bottom). Drawing made by F. Diez-Martín.

Figure S10. Boxplot comparing size (length, breadth and thickness) in natural cobbles (1) and hammerstones (2). Figure made by F. Diez-Martín.

Figure S11. Length and breadth distribution of plain flakes, retouched flakes and large flakes. Figure made by F. Diez-Martín.

Figure S12. Percentage contribution of striking platform types in flakes. Figure made by F. Diez-Martín.

Figure S13. Percentage contribution of dorsal pattern types in flakes.Figure made by F. Diez-Martín.

Figure S14. Percentage contribution of flakes sorted by Toth's types. Figure made by F. Diez-Martín.

Figure S15. Quartz LCT on a thick pseudo large flake (tabular fragment) (181x120x 63 mm, 1399 gr). L6. This tool has been included in the group of distal tips associated to side cutting edges. The two natural areas correspond to the opposed planes of the tabular fragment (0). What it could be considered as lower surface corresponds to the cleavage plane of the blank. The knapping sequence is as follows: 1. On the cleavage plane a continuous series of detachments show step termination due to a second cleavage plane, although broad detachments have been produced (largest negative scar of 38x68 mm).2. Using the previous detachments as a new striking platform, a second series of unipolar and rather invasive detachments (max length 49x 54 mm) produce a rather sinuous left-sided cutting edge and prepares the distal area. 3. An oblique removal in the distal area produces a robust distal tip. 4. Abrupt reduction on the thickest part of the specimen produces a suitable prehensile plane. This series might not be chronologically related to the previously described sequence. Drawing made by F. Diez-Martín.

Figure 16. Quartz LCT on a quartz slab (182x86x56 mm, 1180 gr). L6. A thick abrupt retouched edge is opposed to a rather continuous cutting edge produced via bifacial retouch. Thus, this specimen could fit within the knife type. 1. On the thickest side of the slab, a first abrupt series of lineal detachments is observed. 2. Using as striking platform the opposite edge of the same surface, a bifacial alternating sequence of marginal detachments arranged in dihedral angle, is performed. 3-4. Opposite to the thick backed area, two different series of lineal marginal retouch on both sides, create a bifacial and rather continuous cutting edge associated to a thin distal tip. 5. Proximal abrupt work might be intended to facilitate grasp. This last series might not be chronologically related to the previously described sequence. Drawing made by F. Diez-Martín.

Figure 17. Pointed LCT on a quartz slab (185x109x48 mm, 1269 gr). L6. The two opposed and parallel surfaces are natural planes, although the right edge of the slab shows intense signs of percussion damage. 1. A first unipolar and lineal series of abrupt detachments is produced on the left side of the slab. 2. On the right and thickest side of

the slab, adjacent to the area showing intense signs of pitting, a series of abrupt and short detachments is produced, in order to regularize the distal area. 3. On the same working face of the first unipolar series, an oblique detachment thins out the distal area, anticipating distal tip configuration. 4 A final series of short detachments on the other side creates the final shape of the distal tip. 5 In the basal area of the slab, a series of abrupt detachments has been produced, probably for facilitating grasp. This series might not be chronologically related to the previously described sequence. Drawing made by F. Diez-Martín.

Figure 18. Trihedral pick on a quartz block (188x88x77 mm, 1107 gr). L6. Work on three different planes has created a tool that fits rather well within this type. 1. A first large detachment (54x112 mm) creates one of the three trihedral surfaces. 2. Using the previous negative scar as striking platform, a second series of lineal and preferentially marginal retouch-like detachments is produced on the second face. 3. Opposite to 2 and using the same plane as striking platform (1), a rather invasive series of lineal detachments transforms the third face. 4. On the same surface, a short series of marginal detachments enhances the intersection between faces two and three. 5. The final distal removals are intended distal tip configuration. 6. Basal work creates a proximal pointed area (broken). This last series might not be chronologically related to the previously described sequence. Drawing made by F. Diez-Martín.

Figure 19. Quartz LCT on a thick flake (185x95x60 mm, 1280 gr). L6. Although this specimen formally fits within the knife type (a sinuous cutting edge opposite to an abrupt and thick knapped plane), it represents one of the best examples of unifacial invasive work and crude bilateral symmetry. 1. A first invasive series of large orthogonal or centripetal detachments is observed on the dorsal area (largest negative scar of 54x56 mm). 2. On the left side, a series of abrupt and simple lineal detachments produces a rather rectilinear cutting edge on the distal half of the tool. 3. On the right side of the dorsal area, opposite to 2, and using 1 as striking platform, a series of abrupt detachments is carried out on the thickest part of the blank. 4. Marginal and bifacial detachments enhance the distal convex-pointed area. 5. Proximal bifacial work produced a rather convex basal area. This tool shows, thus, an interesting mixture of features: knife type, invasive dorsal work, crude symmetric outline. Drawing made by F. Diez-Martín.

Figure 20. Quartz LCTs. 1.Slab (147x90x43 mm, 778 gr), L5. Alternate simple and semi-abrupt retouch, quite marginal in occasions, creates a very prominent distal tip. 2. Large flake (160x81x36 mm, 600 gr), L6. Marginal retouch o the dorsal and ventral areas, plus in the distal part of and abrupt side (a longitudinal fracture plane), creates a clear pointed trihedral area. Drawing made by F. Diez-Martín.

Figure 21. Quartz LCT, L6. 1. Slab (156x119x46 mm, 1320 gr). Bifacial removals on the distal area create a sinouous cutting edge. The edges of the slab show negative scars that could be related to bipolar work and signs of percussion. 2. Large and thick quartz flake (164x106x69 mm, 1619 gr). Crude and marginal side and ventral retouch is associated to a distal transversal broad edge. This specimen could be considered as a very crude proto-cleaver. Drawing made by F. Diez-Martín.

Figure 22. Quartz LCTs, L6. 1. Block or fragment (190x55x76 mm, 1072 gr). The intersection of knapping of two surfaces, associated to a third and unmodified natural

surface, creates a rather prominent trihedral area. The distal tip is broken. 2. Fragment (203x74x75 mm, 1620 gr). Abrupt and continuous reduction on two sides (that might imply both bipolar and free hand knapping) create two opposed pointed areas. The interest in creating these massive points is supported by the marginal retouch series observed in the distal pointed area, devoted to final tip enhancement. Drawing made by F. Diez-Martín.

Figure 23. Quartz LCTs, L6. 1. Thick block (155x104x77 mm, 1452 gr). Abrupt detachments and retouch create a distal pointed area associated to a proximal rather convex broad edge. 2. Large flake (148x92x56 mm, 678 gr). Left side lineal knapping on the dorsal surface, ventral work on the proximal area, and distal bifacial retouch create a specimen with a prominent distal tip (broken). Large flake, L6. 3. Quartz large flake (172x56x35 mm, 365 gr). Bulb is prominent on ventral surface, striking platform is natural ant it shows a longitudinal Siret-type fracture on the right side. It probably shows orthogonal arrangements and a few marginal removals on the distal area. Drawing made by F. Diez-Martín.

Figure S24. Radius with green-broken plane. Photo taken by J. Yravedra.

Figure S25. Tibia shaft showing multiple percussion marks in association with green breakage. Photo taken by J. Yravedra.

Figure S26. Humerus shaft from a size 5 animal showing green-broken planes. Photo taken by J. Yravedra.

Figure S27. Impact flake from a megafaunal individual. Photo taken by J. Yravedra.

Figure S28. Percussion mark on the cortical negative scar of a conchoidal fracture. Photo taken by J. Yravedra.

Figure S29. Cut mark on a metapodial shaft. Photo taken by J. Yravedra.

Figure S30. Excavation of the handaxe shown in Figure 5 (Main Text), displayed *in situ.* Photo taken by F. Diez-Martín.

Table captions

Table S1. Distribution of lithics sorted by archaeological level and raw material type.

Table S2. Numeric and percentage contribution of lithics to the various groups of polishing and abrasion considered for this study: R0 (fresh), R1 (slight polishing), R2 (polishing), R3 (intense polishing).

Table S3. Distribution of artifacts sorted by archaeological level and lithic category.

Table S4. Distribution of core types sorted by archaeological level (L= lineal, O= orthogonal, C= centripetal).

Table S5. Distribution of the different types of detached objects sorted by level.

Table S6. Mean size (mm) of plain fakes, retouched flakes, and large flakes retrieved from FLK West.

Table S7. Plain flakes sorted by raw material, striking platform and dorsal pattern.

Table S8. LCTs sorted by morpho-functional group, raw material, blank and retouch types.

Table S9. Measurements of LCTs

Table S10. Distribution of the number of identifiable specimens (NISP) and minimum number of individuals (MNI) per archaeological level documented at FLK West.

Table S11. Frequencies and percentages (parentheses) of bone surface modifications (CM, cut mark; PM, percussion mark; TM, tooth mark) per archaeological level at FLK West. The distribution of marks is also shown according to carcass size (Bunn, 1982). Number of specimens with marks are shown in numerator and total number of specimens per carcass size are shown in denominator.
